# Evaluation of pushing out of children from all English state schools: Administrative data cohort study of children receiving social care and their peers

**DOI:** 10.1016/j.chiabu.2022.105582

**Published:** 2022-05

**Authors:** Matthew A. Jay, Louise Mc Grath-Lone, Bianca De Stavola, Ruth Gilbert

**Affiliations:** a UCL GOS Institute of Child Health, 30 Guilford Street, London WC1N 1EH, United Kingdom; b UCL Institute of Health Informatics, 222 Euston Road, London NW1 2DA, United Kingdom

**Keywords:** Children's social care, Children in need, Off-rolling, Pushing out, Special educational needs, Administrative data

## Abstract

**Background:**

Pushing out (off-rolling) occurs where pupils are illegally excluded from school. Those receiving children's social care (CSC) services (children in need (CiN), on child protection plans (CPPs) or looked after (CLA)) are thought to be at increased risk, but limited evidence inhibits understanding of this phenomenon. The extent of pushing out can be inferred from non-enrolment in administrative data.

**Objective:**

To estimate proportions of children not enrolled across secondary school (aged 11–16, up to year 11) and to explore the association between CSC history and non-enrolment in year 10/11.

**Participants and setting:**

>1 M pupils in year 7 (aged 11/12) in English state schools, 2011/12 and 2012/13.

**Methods:**

We estimated the proportion of children not enrolled across years 8 to 11, disaggregated by CSC history. We assessed with regression modelling the association between CSC history and non-enrolment in years 10/11.

**Results:**

Of children without CSC history, 3.8% had ≥1 year not enrolled by year 11. This was higher in those with a history CiN (8.1%), CPP (9.4%) or CLA (10.4%) status. The odds of non-enrolment in years 10/11 were higher among those with CLA history *vs* non-exposed peers (OR 4.76, 95% CI 4.49–5.05) as well as in those with CPP history (3.60, 3.39–3.81) and CiN history (2.53, 2.49–2.58). History of special educational needs further increased non-enrolment odds, including after confounder adjustment.

**Conclusions:**

Findings imply that children with CSC history are more likely to be pushed out from school than children without, especially those with special educational needs.

## Highlights

• Children with a history of social care are more likely to be unenrolled from school.
• Those with special educational needs and disability are likewise more at risk.
• Legal and regulatory reform is required to protect their right to education.
• Careful use of administrative data can help identify children more at risk.

## 1 Introduction

### 1.1 The importance of education

Educational outcomes for children who receive social care are known to be significantly worse than their peers. Children such as those who have been in foster care are more likely to attain lower grades, are less likely to enter higher education, and are more likely to be absent or excluded (suspended or expelled) from school (Berridge et al., 2020; Department for Education, 2019b; Sebba et al., 2015). The adverse implications of this cannot be understated. Observed links between education and health across life are so persistent over time and place that education has been labelled a “fundamental cause” of inequalities in health (Montez and Friedman, 2015). Higher levels of education are associated with lower risks of mortality (Montez and Friedman, 2015) and morbidity across life, measured using a wide range of indicators including self-rated health (Ljungdahl and Bremberg, 2015; Lynch, 2003; Ross and Wu, 1995), obesity (Hamad et al., 2018; Ljungdahl and Bremberg, 2015), cardiovascular disease (Feldman et al., 1989; Gutzwiller et al., 1989; Yang et al., 2019), depression (Lorant, 2003), cancer (Yang et al., 2019), and others, all of which are major public health issues globally. Besides health, education also gives access to other social resources, such as better paid and better quality employment, and has been associated with social outcomes such as higher social trust, more tolerant attitudes to others, and national development (Hodgson, 1998).

All children have a fundamental human right to education (Hodgson, 1998). Education is recognized as crucial for health and other outcomes and is enshrined in a range of international human rights instruments, including the International Covenant on Economic, Social & Cultural Rights 1966 and the United Nations Convention on the Rights of the Child 1989. This has manifested in many jurisdictions in free and compulsory primary and secondary education. In the United States, for example, education is free and compulsory in all states from age 5 to 7 up to 16 to 18, and most states provide for free high school education up to around 21 years (National Center for Education Statistics, 2017). In England, full-time education is compulsory from age 5 to 16, with most children in England receiving education in state-funded schools (Department for Education, 2021b), and those aged between 16 and 18 must be in some form of education or training (Ford et al., 2016). The state also has particular duties and powers with respect to the welfare, including educational welfare, of children who need children's social care (CSC) services, particularly children looked after (CLA), in recognition of their additional needs (Department for Education, 2018). A failure to meet the needs of such children and protect their right to education can therefore be seen not only as matter of social justice but also through legal and regulatory lenses.

To safeguard the right to education, it is important to understand the extent to which different groups of children may be missing out. It is particularly important to understand how the state protects, or fails to protect, children's right to education when they have a duty to protect children in need of support or at risk of harm (Sullivan and Knutson, 2000). In this paper, we analyze national, linked administrative data which contain longitudinal education and social care records for all children attending state schools in England to identify non-enrolment, which may be an indicator of pushing out—called off-rolling in the UK—among children with and without a history of CSC involvement.

### 1.2 The process and extent of pushing out

In contrast to formal exclusion, whereby legal procedures are followed to exclude (suspend or expel) a child from school, pushing out occurs when a school removes the child from its rolls for some illegitimate purpose, such as to game league tables, avoid costs or make a school appear more desirable to certain families (Owen, 2019; Rosborough, 2009; P. Thomson, 2020). This may particularly affect children with a history of CSC exposure (Ofsted, 2018), who on average achieve poorer results in school exams than their peers. These children are also more likely to have special educational needs and disability (Jay and Gilbert, 2021), particularly social, emotional and mental health problems (Department for Education, 2021a), which, aside from affecting exam performance, require additional resourcing. Schools represent a possible intervention point to improve the education and well-being of these groups of children. However, it is clear that in some instances at least, schools may push out as a way of diminishing demand and disruption from children with additional needs (Department for Education, 2019b; YouGov, 2019).

Evidence from teachers indicates how pushing out occurs. Perceptions were explored qualitatively by YouGov (YouGov, 2019), in which UK teacher respondents reported that off-rolling generally occurs throughout the years immediately before final school exams, and is more likely to occur in schools in deprived areas where maintaining league table performance or high inspection ratings is harder. There was also a perception of “special educational needs and disability (SEND) scapegoating” whereby schools pressure parents to remove their children with SEND to another school or home schooling. This perception that children with SEND are more likely to be off-rolled is supported by evidence from administrative data that children with SEND history are more likely to have unexplained exits from state schools (D. Thomson, 2020). Respondents to the YouGov survey (YouGov, 2019) also reported that teachers are asked to record behavioral incidents of a targeted child for the school's senior leadership team. Parents are then pressured to remove their child from the school either to home or to an alternative placement (Children’s Commissioner for England, 2017; Department for Education, 2019b). The survey, as well as respondents to the Timpson Review of school exclusion (Department for Education, 2019b), collected evidence of school leaders making false representations to parents in threatening them with a formal, permanent exclusion if a child is not removed from the school and stating that such exclusions might appear on records if the child later applied for a job. Respondents suggested that parents with limited understanding and ability to enforce their rights are most at risk of this kind of pressure. Thus, when pushing out/off-rolling occurs, it is in the context of power and informational imbalances between schools and families and involves elements of dishonesty and coercion by schools without effective accountability (Rivkin, 2008; Rosborough, 2009).

Pushing out is an internationally recognized phenomenon, often driven by policy context or specific policies. In the United States, pushing out gained prominence in the 1960s in the context of forcing out students of color from desegregated schools (Rosborough, 2009). In more recent years, test-based accountability of schools under the No Child Left Behind Act is perceived to be a major cause of pushing out (Rosborough, 2009). Test-based accountability, funding frameworks, and the injection of market competition between schools under the schools academization programme, have also been cited in connection with off-rolling in England (Children’s Commissioner for England, 2017; Department for Education, 2019b; House of Commons Education Committee, 2018; P. Thomson, 2020). Specific school policies may also contribute to pushing out where they create an exclusive school environment that fails to meet the complex and diverse needs of children, such as those who need CSC and/or have particular learning needs, who are unable to meet the demands and roles expected of them by the school (Bonell et al., 2019). For example, zero tolerance behavior policies have been cited as a prominent cause of exclusion rates overall as well as their inequitable distribution along socioeconomic and ethnic axes (Skiba, 2000). The same is likely to be true of pushing out, whether schools are acting deliberately to remove a child from the school, or whether the school environment results in the inability of a child to successfully engage in school life.

The true extent of pushing out, and how risk of it varies among children who receive CSC services compared to their peers, is unknown. In England, whole-population administrative data are available on all children enrolled in state schools as well as on their formal exclusions (Jay et al., 2019). There are, however, no official data on pushing out/off-rolling due to its clandestine nature, the same being true in other jurisdictions (Gotbaum, 2002; Rosborough, 2009). Others have attempted to use non-enrolment of particular cohorts in administrative data as a proxy for the practice (Gotbaum, 2002; Nye and Thomson, 2017, Nye and Thomson, 2018; Ofsted, 2018, Ofsted, 2019). These analyses reveal large proportions of children becoming unenrolled from secondary schools, or being discharged from high schools, without explanation. We are aware of no published studies examining non-enrolment/discharge by CSC status in the UK, as shown in a systematic review of research on educational outcomes by CSC status (Jay and Mc Grath-Lone, 2019).

### 1.3 Aims of this study

Using whole-of-England administrative data, we aimed to (1) estimate the proportion of children enrolled in English state schools who ever become not enrolled across secondary school (ages 11–16 years). Because off-rolling is thought to occur especially before final school exams, we also aimed to (2) explore the association between CSC history and non-enrolment in the final two years of compulsory secondary schooling.

## 2 Methods

### 2.1 Cohort and data source

To identify our study cohort, we used the National Pupil Database (NPD)—administrative records for all pupils in state schools in England (Jay et al., 2019). The NPD contains data on, *inter alia*, pupil characteristics and school enrolments for those attending state school. The NPD also contains data on public exam entries (General Certificate of Secondary Education, GCSE, and equivalents) for all pupils, whether attending state or private school. About 7% of all children attend a private school in any one year and, due to transfers into and out of private education, <4% never attend a state school from age 5 to 16 (Anders et al., 2020; Department for Education, 2021b).

All children in the cohort had to be enrolled in state school in year 7 (age 11/12), which is the start of secondary schooling, in 2011/12 or 2012/13 (academic years: September to August). We analyzed separate cohorts attending mainstream schools and special schools (1.6% of state school children) in year 7. Both cohorts were followed up through the state education system in England until the end of year 11 (age 15/16). Children were identified as being in year 7 using a variable in the NPD indicating this. Some children were not designated as following any school year. Such children were included in the analysis if they were aged 11 at the start of the 2011/12 or 2012/13 academic years.

The small number (0.2%) of children attending Alternative Provision (AP) and Pupil Referral Units (PRUs) (Department for Education, 2016) were included in the mainstream school cohort if dually-enrolled or only attending an AP/PRU. Those dually enrolled in an AP/PRU and special school were analyzed in the special school cohort.

We minimized the risk of misclassifying non-enrolled children as off-rolled by restricting the cohort to children who were enrolled in state school in year 7, the first year of secondary school. In England, children attend primary school from year 1 to 6 (aged 5 to 11) and transition to secondary school in year 7, aged 11. Preliminary analyses revealed that, when selecting children based on enrolment at the start of primary school (age 5 to 6), 2% became unenrolled in year 7 and did not return to the state school sector, which was the second most common enrolment pattern after complete enrolment in every year (Supplementary File 1, Tables S1.4 and S1.5). These are likely to have been children who transferred to private school. Therefore, specifying that a child must be enrolled in year 7 to be in the cohort minimized the risk of including children who transferred to private schooling, which, because the NPD does not collect data on private school enrolments, is an outcome indistinguishable from off-rolling.

In order to identify children with a history of CSC exposure we used the children in need (CiN) census (Emmott et al., 2019) and CLA dataset (Mc Grath-Lone et al., 2016). These are longitudinal, all-of-England, administrative datasets that include data on, respectively, all children in England who become CiN (including those allocated a child protection plan (CPP)) and all children who become CLA. As the CiN census began in October 2008, CiN data were available from year 3 (aged 7/8) or 4 (aged 8/9), depending on whether the child was in year 7 in 2011/12 or 2012/13. The CLA dataset is linkable to the NPD from 2005, meaning data were available from the reception year (aged 4/5) or year 1 (aged 5/6). However, we measured CiN, CPP and CLA exposure from year 4 for all children to ensure consistency in exposure measurement.

Data were linked using the nationally unique anonymized Pupil Matching Reference (PMR).

The means by which we derived the cohorts and cleaned the data are presented in Supplementary File 2, along with a flow diagram in Fig. S2.1.

### 2.2 Study design

**Fig. 1:**
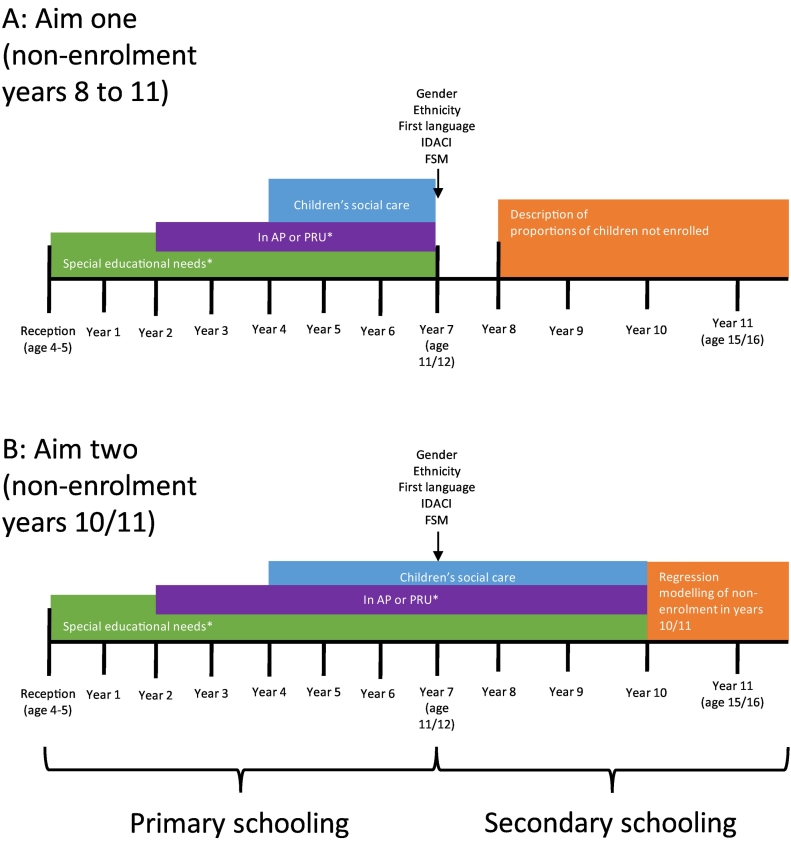
Schematic overview of analyses. Data from the same cohort of children were used to address both aims, namely children aged 11/12 at the start of academic year 7 in 2011/12 or 2012/13 (or aged 11 at the start of those academic years if designated as following any year). * Coverage varies for each cohort. See Table 1 for details. FSM free school meals; IDACI income deprivation affecting children index.

To estimate what proportion of children who became non-enrolled (first aim), we examined the proportions of children not enrolled in years 8 to 11 (aged 12 to 16) by CSC exposure in primary school (from years 4 to 6, aged 8 to 11; Fig. 1A). CSC exposure was measured using four exclusive, hierarchical groups across primary school years 4 to 6 (ages 8 to 11; Table 1). Children who received different levels of CSC support across this period were categorized into the most intensive level of CSC provision that they received. Services for CiN were the lowest level, followed by being subject to a CPP and finally being a CLA (see Supplementary File 2 for full definitions).

The outcome, non-enrolment in a school year from year 8 to 11, was defined by absence of the child's record in the spring school, AP or PRU census files for the relevant years. For example, a child enrolled in year 7 in 2011/12 was not enrolled in year 8 if their PMR could not be found in the 2012/13 census files. Likewise, a child enrolled in year 7 in 2012/13 was not enrolled in year 8 if their PMR was not found in the 2013/14 census files. Repeating a year (grade retention) is rare in England (Education Endowment Foundation, 2018). As all children had to be enrolled in year 7 to be included in the cohort, non-enrolment in year 7 was not examined.

**Table 1:** Sociodemographic variables used in the analyses

Variable	Categories	Details
CSC exposure	None, CiN, CPP, CLA	Measured across year 4 to 6 (Aim 1) or 4 to 9 (Aim 2). These were hierarchical, mutually exclusive groups and children were categorized according to the highest status they experienced.
Gender	Female, Male*	Measured at year 7.
Ethnicity	White, Black, Mixed, Asian, Other	Measured at year 7.
First language	English, Other	Measured at year 7.
IDACI (2015 version)	Fifths ranging from 1 (most deprived) to 5 (least deprived)	IDACI is an area-based measure of income deprivation (the proportion of children in households whose parents claim benefits) at the neighborhood level (average of 1500 people). Measured at year 7.
FSM claimed	Claimed & eligible (1), or not (0)	FSM is available for children in households where the parent(s) claim certain benefits. Measured at year 7.
IDACI/FSM	1,1 (most deprived IDACI, FSM claimed) 1,0 (most deprived, FSM not claimed) 2,1 2,0 … 5,1 (least deprived, FSM claimed) 5,0 (least deprived, FSM not claimed)	Measured at year 7.
Region	Former Government Office Regions: East Midlands, East of England, London, North East, North West, South East, South West, West Midlands, Yorkshire & The Humber	Measured at year 7.
SEND provision	None or Any	Measured from reception (2012/13 cohort) or year 1 (2011/12 cohort) to year 6 or 9.
Highest SEND provision	None, AAPS, SEHCP	Measured from reception (2012/13 cohort) or year 1 (2011/12 cohort) to year 6 or 9.
In AP/PRU	Yes or No	Due to data availability, AP attendance was measured from year 2 (2012/13 cohort) or year 3 (2011/12 cohort) to year 6 or 9. Similarly, PRU attendance was measured from year 4 (2012/13 cohort) or year 5 (2011/12 cohort) to year 6 or 9.

AAPS Action, Action Plus or Support; AP alternative provision; CiN child in need; CLA child looked after; CPP child protection plan; CSC children’s social care; FSM free school meals; IDACI income deprivation affecting children index; PRU pupil referral unit; SEND special educational needs and disability; SECHP statement or Education, Health and Care Plan. * Male and female are the only available categories in the National Pupil Database.

We addressed our second aim (exploring the association between CSC history and non-enrolment in the final two years of schooling) by comparing children in contact with CSC in years 4 to 9 and their enrolment status in years 10/11 (Fig. 1B). In these analyses, a child was not enrolled if they met two criteria: (1) they were not enrolled in year 10 or 11; and (2) sat <5 GCSE or equivalent exams in year 11, determined from the Key Stage 4 exams NPD data module, which also contains data on children sitting these exams in private schools (Jay et al., 2019). This outcome is referred to as non-enrolment in years 10/11. The second criterion was specified to rule out children who sit GCSE exams in other settings and therefore were still receiving an education. The choice of five GCSEs as a threshold for indicating participation in education outside the state sector was based on historic school accountability measures (Leckie and Goldstein, 2017) on the rationale that this threshold represents a bare minimum that would have reflected an effective education (the legal requirement for all children (Ford et al., 2016)).

We extracted sociodemographic variables from the NPD census files on pupils' gender, ethnicity, first language, history of SEND provision, the area-based income deprivation affecting children index (IDACI, (Ministry of Housing Communities & Local Government, 2015)), whether claiming free school meals (FSM), available for low income families (HM Government, 2015), and geographical region (Table 1).

### 2.3 Statistical methods

To address our first aim, we calculated the annual proportion of children in the cohort who were not enrolled in each of academic years 8, 9, 10 and 11. Next, we calculated the cumulative proportion who were not enrolled by years 8, 9, 10 and 11. These proportions were disaggregated by CSC exposure (years 4 to 6) and the other sociodemographic variables.

To determine the association between CSC exposure and non-enrolment in years 10/11 (second aim), we calculated the proportion of non-enrolment in years 10/11 overall and by CSC exposure (measured academic years 4 to 9) and the other sociodemographic variables. We then used 3-level hierarchical logistic regression with random intercepts specified for local authorities and regions to account for geographical clustering. We first estimated a series of univariable models where each model had as its sole predictor each variable. We then estimated a series of models in the following order. After fitting a model with no explanatory variables (empty model), CSC exposure (years 4 to 9) was added first on its own and then adjusted for SEND history. We used a binary variable of ever having any SEND, rather than a more detailed variable that described different levels of SEND provision, so that the models could converge. The adjustment was motivated by the fact that there is a high incidence of SEND provision among CLA and CiN (Jay and Gilbert, 2021) and there was an expectation that such provision was associated with non-enrolment (Ofsted, 2019; D. Thomson, 2020; YouGov, 2019). We then entered an interaction term between these two variables. We hypothesized that a statistically significant interaction between CSC and SEND provision could reflect improved outcomes, due to additional support, or worse outcomes, due to more complex need for services.

Next, we adjusted the model for possible confounders: gender, ethnicity, first language, the combined IDACI/FSM variable and whether the child had been enrolled in AP/PRU up to year 9 (Table 1). These variables were included as confounders because exploratory analyses showed that ethnicity, language, IDACI/FSM and being in an AP/PRU were all associated with becoming non-enrolled. Ethnicity and deprivation have also been shown to be associated with CSC exposure (Bywaters et al., 2018; Mc Grath-Lone et al., 2015, Mc Grath-Lone et al., 2017) and we considered it not unreasonable to expect that language would also be correlated. Gender did not appear to be associated with non-enrolment in our exploratory analyses. However, gender is correlated with SEND (Department for Education, 2020) and so this variable may have been relevant in the CSC-SEND interaction. Being in an AP or PRU is indicative of additional underlying behavioral or health needs (Department for Education, 2016). These factors, too, are likely confounders in the association between CSC and off-rolling.

The amount of between-local authority and between-region variation explained by variables in the models was assessed by calculating the percentage change between models in the local authority-level and region-level random intercepts' standard deviation.

All analyses were conducted using R 3.6.2 with the following packages: *data.table* (Dowle and Srinivasan, 2021), *plyr* (Wickham, 2011), *tidyR* (Wickham and Henry, 2021), *reshape2* (Wickham, 2007), *tableone* (Kazuki et al., 2020), *ggplot2* (Wickham and Chang, 2021), *gridExtra* (Auguie, 2017), *lme4* (Bates et al., 2015) and *lmtest* (Zeiles, 2020).

### 2.4 Ethics and data protection

We obtained all relevant ethical and governance approvals as detailed in Supplementary File 2. Statistical disclosure controls were applied such that cell counts <10 are suppressed.

## 3 Results

### 3.1 Characteristics

There were 1,081,779 children in total (545,942 in the 2011/12 cohort and 535,837 in the 2012/13 cohort). There was a total of 5469 (0.5%) children who had missing data on gender, ethnicity, language or IDACI. No other variable had missing data. Because the number and overall proportion of children with missing data was negligible (Supplementary File 3), we proceeded by listwise deletion. Of the 1,076,310 children in the cohort with complete data, 16,529 (1.6%) were enrolled in special schools in year 7 and the remaining 1,059,781 were in mainstream schools.

The characteristics of the children in the 2011/12 and 2012/13 cohorts are presented separately in Supplementary File 4. Any differences between the cohorts were negligible and, as there were no known theoretical reasons why the two cohorts should have been treated differently, all children were analyzed as one cohort. The characteristics of the cohort of 1,059,781 children in mainstream settings are given in Table 2.

A total of 76,517 (7.2%) children had some form of CSC exposure across years 4 to 6 (Table 2). The largest group was the CiN group (*n* = 65,880, 6.2%). In the CPP group were 5202 (0.5%) children and in the CLA group, 5435 (0.5%). Measured across years 4 to 9, 120,454 (11.4%) children had some form of CSC exposure (Table 2). By year 9, the CiN group had 96,306 (9.1%) children, the CPP group 12,987 (1.2%) and the CLA group, 11,161 (1.1%).

**Table 2:** Characteristics of the mainstream schools' cohort (*n* = 1,059,781 children enrolled in year 7 in 2011/12 or 2012/13).

		n (%)
n		1,059,781
CSC exposure	None	983,264 (92.8%)
(aim one: yr 4 to 6)	CiN	65,880 (6.2%)
	CPP	5202 (0.5%)
	CLA	5435 (0.5%)

CSC exposure	None	939,327 (88.6%)
(aim two: yr 4 to 9)	CiN	96,306 (9.1%)
	CPP	12,987 (1.2%)
	CLA	11,161 (1.1%)

Female		520,945 (49.2%)

Ethnicity	White	843,005 (79.5%)
	Black	54,195 (5.1%)
	Mixed	44,346 (4.2%)
	Asian	10,3186 (9.7%)
	Other	15,049 (1.4%)

First language not English		167,921 (15.8%)

IDACI fifths	1 (most deprived)	251,490 (23.7%)
	2	219,771 (20.7%)
	3	201,771 (19%)
	4	194,997 (18.4%)
	5 (least deprived)	191,752 (18.1%)

FSM claimed	Yes (1)	190,568 (18%)

IDACI/FSM	1,1	94,913 (9%)
	1,0	156,577 (14.8%)
	2,1	49,144 (4.6%)
	2,0	170,627 (16.1%)
	3,1	25,822 (2.4%)
	3,0	175,949 (16.6%)
	4,1	14,046 (1.3%)
	4,0	180,951 (17.1%)
	5,1	6643 (0.6%)
	5,0	185,109 (17.5%)

Region	East Midlands	92,537 (8.7%)
	East of England	121,208 (11.4%)
	London	151,514 (14.3%)
	North East	51,267 (4.8%)
	North West	145,995 (13.8%)
	South East	168,232 (15.9%)
	South West	102,075 (9.6%)
	West Midlands	118,687 (11.2%)
	Yorkshire & The Humber	108,266 (10.2%)

Ever SEND	(primary school to yr 6)	373,741 (35.3%)
Highest ever SEND	None	686,040 (64.7%)
(primary school to yr 6)	AAPS	353,689 (33.4%)
	SEHCP	20,052 (1.9%)

Ever SEND	(primary school to yr 9)	426,172 (40.2%)
Highest ever SEND	None	633,609 (59.8%)
(primary school to yr 9)	AAPS	398,079 (37.6%)
	SEHCP	28,093 (2.7%)

In AP/PRU (primary school to yr 6)		796 (0.1%)

In AP/PRU (primary school to yr 9)		6330 (0.6%)

Where not stated, variables were recorded at year 7 (aged 11/12). AAPS Action, Action Plus or Support; AP/PRU Alternative provision / Pupil Referral Unit; CiN child in need; CLA child looked after; CPP child protection plan; CSC children’s social care; FSM free school meals (family-level measure of income deprivation); IDACI income deprivation affecting children index (area-based measure of income deprivation); SEHCP statement or Education, Health & Care Plan; SEND special educational needs and disabilities; yr year.

### 3.2 Non-enrolment in years 8 to 11

**Fig. 2:**
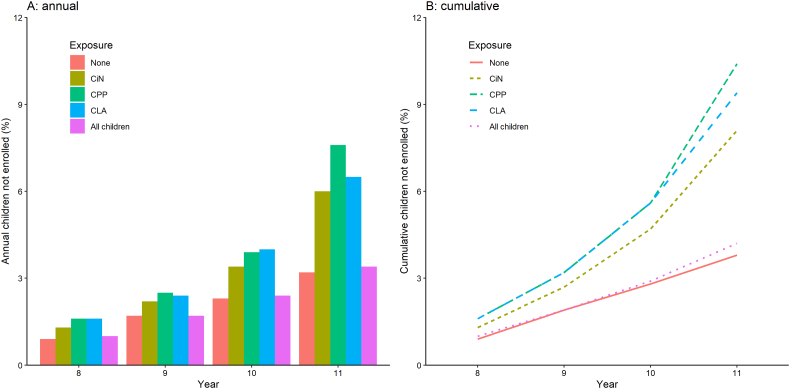
Annual and cumulative proportions of children not enrolled across years 8 to 11. Data in tabular form are available in Supplementary File 5. CiN children in need; CLA children looked after; CPP child protection plan. Annual and cumulative proportions of children not enrolled across years 8 to 11. Data in tabular form are available in Supplementary File 5. CiN children in need; CLA children looked after; CPP child protection plan.

Fig. 2A shows the proportions of children in the mainstream cohort who were not enrolled in each academic year. Among all children, the yearly proportion rose steadily from 1.0% in year 8 to 3.4% in year 11. The same pattern was observed in children without any CSC exposure. However, among the three groups with CSC exposure, all of whom had higher rates of non-enrolment each year compared with their non-exposed peers, there was a sharp increase in the proportion not enrolled from year 10.

Fig. 2B shows the cumulative proportion of non-enrolment, *i.e.*, the cumulative proportion of children in the cohort who were ever not enrolled in year 8, 9, 10 and, finally, 11. As with the annual proportions, the cumulative proportion of non-enrolment was higher in the CSC-exposed children compared to the non-exposed. The highest by year 11 was in the CPP group (10.4%), followed by CLA (9.4%), CiN (8.1%) and children with no CSC contact in years 4 to 6 (3.8%). An uptick in years 10 and 11 of non-enrolment in the CSC-exposed children—but not in the unexposed—can also be seen in Fig. 2B.

The annual and cumulative proportions of non-enrolment are presented in tabular form in Supplementary File 5, disaggregated by: CSC exposure, gender, ethnicity, first language, IDACI, FSM, IDACI/FSM, region, SEND history and attendance at AP/PRU.

### 3.3 Risk factors for non-enrolment in years 10/11

Table 3 shows the number and proportions of children experiencing non-enrolment in years 10/11 (as well the separate criteria constituting the outcome definition). Overall, 31,283 (3.0%) of all children were not enrolled in years 10/11. The proportions of non-enrolment among children exposed to CSC in years 4 to 9 were higher: CiN, 6.0%; CPP, 8.4%; CLA, 10.7%.

**Table 3:** Number and proportion of children in the mainstream schools' cohort (n = 1,059,781) who were not enrolled in year 10/11, who sat <5 GCSE or equivalent exams and who were either not enrolled or sat <5 exams.

		Not enrolled yr 8/9	Not enrolled yr 10/11	Sat <5 GCSEs or equivalents	**Not enrolled yr 10/11 & <** **5 GCSEs or equivalents**
		n (%)	n (%)	n (%)	**n (%)**
All children		20,593 (1.9%)	39,228 (3.7%)	70,045 (6.6%)	**31,283 (3.0%)**

CSC exposure	None	17,049 (1.8%)	29,995 (3.2%)	44,404 (4.7%)	**23,209 (2.5%)**
(yr 4 to 9)	CiN	2582 (2.7%)	6712 (7.0%)	18,030 (18.7%)	**5790 (6.0%)**
	CPP	402 (3.1%)	1200 (9.2%)	3888 (29.9%)	**1086 (8.4%)**
	CLA	560 (5.0%)	1321 (11.8%)	3723 (33.4%)	**1198 (10.7%)**

Gender	Male	10,545 (2.0%)	20,114 (3.7%)	40,641 (7.5%)	**16,286 (3.0%)**
	Female	10,048 (1.9%)	19,114 (3.7%)	29,404 (5.6%)	**14,997 (2.9%)**

Ethnicity	White	14,058 (1.7%)	29,783 (3.5%)	56,546 (6.7%)	**23,839 (2.8%)**
	Black	1917 (3.5%)	2827 (5.2%)	4027 (7.4%)	**2293 (4.2%)**
	Mixed	1140 (2.6%)	2076 (4.7%)	3836 (8.7%)	**1676 (3.8%)**
	Asian	2565 (2.5%)	3286 (3.2%)	4151 (4.0%)	**2373 (2.3%)**
	Other	913 (6.1%)	1256 (8.3%)	1485 (9.9%)	**1102 (7.3%)**

Language	English	14,248 (1.6%)	30,304 (3.4%)	58,948 (6.6%)	**23,862 (2.7%)**
	Not English	6345 (3.8%)	8924 (5.3%)	11,097 (6.6%)	**7421 (4.4%)**

IDACI fifths	1 (most deprived)	5105 (2.0%)	11,580 (4.6%)	25,710 (10.2%)	**9789 (3.9%)**
	2	4283 (1.9%)	8480 (3.9%)	16,445 (7.5%)	**7113 (3.2%)**
	3	3745 (1.9%)	6908 (3.4%)	11,841 (5.9%)	**5576 (2.8%)**
	4	3531 (1.8%)	6138 (3.1%)	8840 (4.5%)	**4577 (2.3%)**
	5 (least deprived)	3929 (2.0%)	6122 (3.2%)	7209 (3.8%)	**4228 (2.2%)**

FSM	No (0)	15,892 (1.8%)	27,864 (3.2%)	42,568 (4.9%)	**21,398 (2.5%)**
claimed	Yes (1)	4701 (2.5%)	11,364 (6.0%)	27,477 (14.4%)	**9885 (5.2%)**

IDACI/	1,1 (most deprived & claimed FSM)	2243 (2.4%)	5816 (6.1%)	14,583 (15.4%)	**5013 (5.3%)**
FSM	1,0	2862 (1.8%)	5764 (3.7%)	11,127 (7.1%)	**4776 (3.1%)**
	2,1	1246 (2.5%)	2955 (6.0%)	6864 (14.0%)	**2619 (5.3%)**
	2,0	3037 (1.8%)	5525 (3.2%)	9581 (5.6%)	**4494 (2.6%)**
	3,1	673 (2.6%)	1483 (5.7%)	3545 (13.7%)	**1282 (5.0%)**
	3,0	3072 (1.7%)	5425 (3.1%)	8296 (4.7%)	**4294 (2.4%)**
	4,1	364 (2.6%)	773 (5.5%)	1759 (12.5%)	**675 (4.8%)**
	4,0	3167 (1.8%)	5365 (3.0%)	7081 (3.9%)	**3902 (2.2%)**
	5,1	175 (2.6%)	337 (5.1%)	726 (10.9%)	**296 (4.5%)**
	5,0 (least deprived & no FSM)	3754 (2.0%)	5785 (3.1%)	6483 (3.5%)	**3932 (2.1%)**

Region	East Midlands	1618 (1.7%)	3227 (3.5%)	5824 (6.3%)	**2601 (2.8%)**
	East of England	2320 (1.9%)	4414 (3.6%)	7863 (6.5%)	**3565 (2.9%)**
	London	4173 (2.8%)	7246 (4.8%)	11,693 (7.7%)	**5929 (3.9%)**
	North East	688 (1.3%)	1440 (2.8%)	3309 (6.5%)	**1141 (2.2%)**
	North West	2273 (1.6%)	4534 (3.1%)	9857 (6.8%)	**3680 (2.5%)**
	South East	3740 (2.2%)	6839 (4.1%)	10,722 (6.4%)	**5416 (3.2%)**
	South West	1986 (1.9%)	3803 (3.7%)	6698 (6.6%)	**2990 (2.9%)**
	West Midlands	2036 (1.7%)	3870 (3.3%)	7180 (6.0%)	**3099 (2.6%)**
	Yorkshire & The Humber	1759 (1.6%)	3855 (3.6%)	6899 (6.4%)	**2862 (2.6%)**

AP/PRU	No	20,145 (1.9%)	38,255 (3.6%)	65,663 (6.2%)	**30,392 (2.9%)**
(to yr 9)	Yes	448 (7.1%)	973 (15.4%)	4382 (69.2%)	**891 (14.1%)**

Ever SEND	No	114,74 (1.8%)	18,349 (2.9%)	20,696 (3.3%)	**13,641 (2.2%)**
(to yr 9)	Yes	9119 (2.1%)	20,879 (4.9%)	49,349 (11.6%)	**17,642 (4.1%)**

Highest	None	11,474 (1.8%)	18,349 (2.9%)	20,696 (3.3%)	**13,641 (2.2%)**
ever SEND	AAPS	8491 (2.1%)	19,459 (4.9%)	40,050 (10.1%)	**16,339 (4.1%)**
(to yr 9)	SEHCP	628 (2.2%)	1420 (5.1%)	9299 (33.1%)	**1303 (4.6%)**

The final column in bold represents the variable used as the outcome in the regression modelling. AAPS Action, Action Plus or Support; AP/PRU Alternative provision / Pupil Referral Unit; CiN child in need; CLA child looked after; CPP child protection plan; CSC children’s social care; FSM free school meals (family-level measure of income deprivation); GCSE General Certificate of Secondary Education; IDACI income deprivation affecting children index (area-based measure of income deprivation); SEHCP statement or Education, Health & Care Plan; SEND special educational needs and disabilities; yr year.

Hierarchical logistic regression results are presented in Table 4 (full results in Supplementary File 6). Model 2 shows that the odds of the non-enrolment were 4.76 (95% CI 4.49, 5.05) times higher among the CLA group than the non-exposed group; this factor for the CPP group was 3.60 (95% CI 3.39, 3.81) times and for the CIN group, 2.53 (95% CI 2.49, 2.58) times. These odds ratios (ORs) were reduced when adjusted for SEND but remained statistically significant (model 3).

**Fig. 3:**
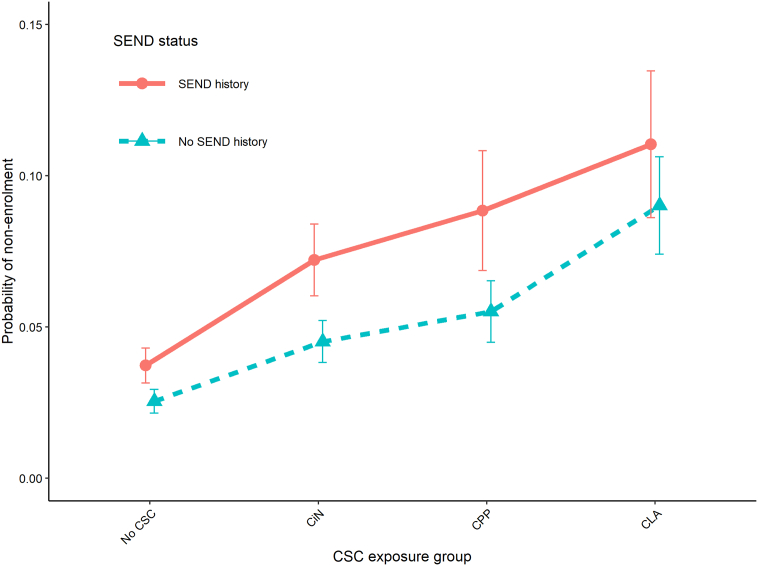
Predicted probabilities of non-enrolment in years 10/11 by social care status and special educational needs and disability status derived from Model 5 (Table 3). Dots represent the predicted probability of non-enrolment in years 10/11 (and sitting <5 GCSE exams) as calculated from the coefficients in Model 5 (Table 4 and Supplementary File 6). Error bars are 95% confidence intervals. Values for other variables in the model were set to their reference categories. CiN children in need; CLA children looked after; CPP child protection plan; GCSE General Certificate of Secondary Education; SEND special educational needs and disability; yr year. Predicted probabilities of non-enrolment in years 10/11 by social care status and special educational needs and disability status derived from Model 5 (Table 3). Dots represent the predicted probability of non-enrolment in years 10/11 (and sitting <5 GCSE exams) as calculated from the coefficients in Model 5 (Table 4 and Supplementary File 6). Error bars are 95% confidence intervals. Values for other variables in the model were set to their reference categories. CiN children in need; CLA children looked after; CPP child protection plan; GCSE General Certificate of Secondary Education; SEND special educational needs and disability; yr year.

There was evidence from model 4 that SEND interacted with being exposed to CSC, such that CSC was associated with higher odds of non-enrolment where SEND provision had been made at some point before year 9 (Table 4). This interaction was statistically significant (*p* = 0.005) and persisted after adjusting for confounders (model 5 in Table 4). The estimated probability of non-enrolment in each of the exposure groups is shown in Fig. 3 where the increase in the risk of non-enrolment with a history of SEND was more pronounced in the CiN and CPP groups than in the CLA group.

**Table 4:** Odds ratios (OR) and 95% confidence intervals (CI) from hierarchical logistic regression models of non-enrolment in years 10/11 of children in the mainstream cohort.

			Model				
			1 (empty)	2	3	4	5*
CSC exposure (yr 4 to 9)	None		··	Reference	Reference	Reference	Reference
	CiN	OR (95% CI)		2.53 (2.49, 2.58)	2.23 (2.14, 2.31)	2.10 (1.98, 2.22)	1.82 (1.72, 1.93)
	CPP	OR (95% CI)		3.60 (3.39, 3.81)	3.06 (2.89, 3.25)	2.72 (2.42, 3.06)	2.23 (1.98, 2.50)
	CLA	OR (95% CI)		4.76 (4.49, 5.05)	3.86 (3.64, 4.09)	4.57 (4.06, 5.14)	3.82 (3.40, 4.30)
SEND to yr 9		OR (95% CI)			1.63 (1.60, 1.66)	1.62 (1.58, 1.65)	1.49 (1.46, 1.52)
Interaction	CiN & SEND	OR (95% CI)				1.08 (1.02, 1.15)	1.09 (1.03, 1.16)
	CPP & SEND	OR (95% CI)				1.14 (1.01, 1.28)	1.12 (0.97, 1.28)
	CLA & SEND	OR (95% CI)				0.82 (0.73, 0.92)	0.84 (0.73, 0.96)

Variance components							
Level 2 (LA) SD			0.24	0.22	0.22	0.22	0.22
% explained			..	8.3%	8.3%	8.3%	8.3%
Level 3 (region) SD			0.18	0.18	0.18	0.18	0.15
% explained			..	0%	0%	0%	16.7%

Model summaries							
AIC			280,310	274,787	273,104	273,097	268,991
LRT *p* value†			··	<0.001	<0.001	0.005	<0.001

The outcome was non-enrolment in year 10/11 and sitting <5 GCSE or equivalent exams (final column of Table 3). In all models there were 1,059,781 children in 151 LAs in 9 regions. See Supplementary File 6 for full model results. See Fig. 3 for a graphical representation of the interaction term in Model 5. The % variation explained is calculated as the relative difference between each model's level 2 and 3 random intercepts' standard deviations and those of Model 1. * Adjusted for gender, ethnicity, first language, IDACI/FSM, in AP/PRU to year 9. † LRTs were conducted against the previous model. AIC Akaike Information Criterion; CI confidence interval; CiN child in need; CLA child looked after; CPP child protection plan; CSC children’s social care; GCSE General Certificate of Secondary Education; LRT likelihood ratio test; OR odds ratio; SD standard deviation; SEND special educational needs and disability.

The outcome was non-enrolment in year 10/11 and sitting <5 GCSE or equivalent exams (final column of Table 3). In all models there were 1,059,781 children in 151 LAs in 9 regions. See Supplementary File 6 for full model results. See Fig. 3 for a graphical representation of the interaction term in Model 5. The % variation explained is calculated as the relative difference between each model's level 2 and 3 random intercepts' standard deviations and those of Model 1. * Adjusted for gender, ethnicity, first language, IDACI/FSM, in AP/PRU to year 9. † LRTs were conducted against the previous model. AIC Akaike Information Criterion; CI confidence interval; CiN child in need; CLA child looked after; CPP child protection plan; CSC children’s social care; GCSE General Certificate of Secondary Education; LRT likelihood ratio test; OR odds ratio; SD standard deviation; SEND special educational needs and disability.

### 3.4 Children in special schools

The analyses for the cohort of 16,529 children who were enrolled in special schools in year 7 are presented in Supplementary File 7. Children in contact with CSC in years 4 to 6 were more likely to be non-enrolled than their peers, annually and cumulatively, across years 8 to 11. Overall, 4.6% of the special schools' cohort were not enrolled at least once by year 11 (Table S7.2). The proportions by CSC status were: no exposure, 3.3%: CiN, 5.3%; CPP, 8.2%; CLA 10.0% (Table S7.2). Children in contact with CSC in years 4 to 9 were also more likely to be non-enrolled than their peers in years 10/11 after adjusting for confounding variables: compared to those without any CSC exposure, the odds of non-enrolment among the CiN group were 1.51 (95% CI 1.24, 1.83) higher; those in the CPP group, 3.25 (95% CI 2.33, 4.54) higher; and those in the CLA group 3.49 (95% CI 2.71, 4.50) times higher.

## 4 Discussion

### 4.1 Summary

We found that children with a history of CSC involvement were significantly more likely to leave the English state school sector compared with their peers, especially in the final two mandatory years of secondary school education. We also observed that children with SEND were likewise more likely to become unenrolled. These two factors interacted in such a fashion that children with CSC history, especially CiN and those subject to a CPP, were at additional risk of non-enrolment. These relationships persisted when adjusting for confounders. Non-enrolment in years 10/11 and sitting <5 GCSE or equivalent is likely to be due to pushing out/off-rolling. These findings imply that pushing out/off-rolling disproportionately affects vulnerable children with social welfare and additional learning needs, which in turn may contribute to educational and health inequalities across life.

Children in the CLA and CPP groups had similar rates of non-enrolment both cumulatively (Fig. 2) and in years 10/11 (Table 3), differing only by one or two percentage points. This is perhaps unsurprising given that the threshold for both a CPP and a care order is the same: risk or experience of significant harm (Supplementary File 2). It might be expected that CLA have a much higher risk of non-enrolment given higher levels of underlying need and perhaps more domestic instability (Office of the Children’s Commissioner, 2021). However, not all children become CLA on the basis of abuse or neglect and there is significant heterogeneity in the experience of CLA (Mc Grath-Lone et al., 2020). Additionally, there may have been a protective effect of the care system and the additional support that CLA receive (such as in Personal Education Plans and from Virtual School Heads and designated teachers (Department for Education, 2018)) which reduced the risk of non-enrolment among children who become looked after.

CLA with a history of SEND provision did not have a statistically significantly higher risk of non-enrolment in years 10/11 than CLA without (Fig. 3). SEND provision may have had less of an impact in the CLA group compared to the other groups due to higher levels of underlying need. However, it cannot be ruled out that this was a statistical artifact that would be removed by a larger study with more precision. Further, future research should seek to investigate the effects of unmet SEND need, which may increase the risk of non-enrolment among children without recorded SEND need.

To our knowledge, this is the first study to attempt to quantify pushing out/off-rolling in this way among children who receive CSC services in England. It is not therefore possible to compare our results directly with previous research except to note that our findings are consistent with reported anecdote (YouGov, 2019) and the suggestion by the Office for Standards in Education (Ofsted) in their blog post that CLA are more at risk of becoming non-enrolled (Ofsted, 2018, Ofsted, 2019).

### 4.2 Limitations and strengths

Non-enrolment does not conclusively indicate that a child is not receiving an education. Possible alternatives include emigration and death. An average of 15,000 (approximately 0.1%) persons aged under 15 left the UK between 2011 and 2016 per year (data for school-aged children only leaving the UK are not available (Office for National Statistics, 2020c)). In the year ending 2014, 5214 children aged 11 to 15 (about 0.2% of all children of these ages) left England for one of the other three UK countries (Office for National Statistics, 2020b). The mortality rate among 5–15 year-olds in England in 2015 was 9.3 per 100,000 (Office for National Statistics, 2020a). Emigration and death are therefore unlikely to account for a substantial proportion of our findings.

It is likely that few children in contact with CSC became not enrolled because they transferred to home or private schooling. Firstly, approximately 45,000 to 50,000 children were home-schooled in 2017 (ADCS, 2017; Department for Education, 2019a) or 0.7% of the school-aged population. This figure includes children home-schooled from the outset. Secondly, although it may be that some children with CSC history attend private schools, only 7% of all children are enrolled in private schools each year and fewer than 4% of all children are never in state education from age 5 to 16 in England (Anders et al., 2020; Department for Education, 2021b). It is therefore unlikely that transfer to private schooling is widespread among children with CSC history.

However, transfer to private education could not be entirely ruled out. To minimize the possibility that our analyses of non-enrolment in years 10/11 included children who transferred to private school, we stipulated that the child must both be unenrolled (either in year 10 or 11) and sit <5 GCSEs or equivalents in year 11. Children educated in private and home settings would normally sit at least five, if not ten or more, exams and would thus appear in the NPD GCSE data. Therefore, our outcome definition ensures that children in these other settings are not counted as pushed out/off-rolled children. As it is reasonable to expect that children in contact with CSC are less likely to transfer to private schooling, and possibly to home school, than children from more advantaged and wealthier backgrounds, any bias due to such transfers will predominately affect children not in contact with CSC, and thereby may have attenuated the odds ratios reported for comparisons of CSC-exposed children with those not exposed.

We may have misclassified some children as not enrolled in education who were in home or private schooling and who did not sit GCSEs. A small proportion of children may not have taken exams because of low academic ability, which may itself be a consequence of past trauma. A lower rate of exam entry, as observed in the groups of children in our study who had CSC history, could be seen as an unmet need, one that is especially concerning given the importance of final school exams for future education and job prospects, and particularly in situations where a child is not enrolled in compulsory education.

Another explanation for non-enrolment could be that children may have permanently absconded from school and the school was unable to locate them. It is not possible to determine from the data the extent to which this may have happened in our cohort. However, it should be noted that such an outcome is likely as deleterious as pushing out given that it represents a failure by the state to retain children in education and represents a potential safeguarding issue. This is especially so concerning those known to CSC.

Another limitation is that we can only count all children in the spring term (*i.e.*, the January to March term in the middle of the academic year) as it is only then that children in all state schools are included in the NPD. This means that we could not identify children temporarily not enrolled in the spring term who were enrolled in the autumn or summer terms. Further, the crude measures of income deprivation (area-based IDACI and family-based FSM) likely under-estimate the effects of these exposure. For example, IDACI is an average area measure based on proportions of individuals in receipt of certain benefits, not all those eligible. Likewise, approximately 14% of pupils eligible for FSM, based on parental receipt of benefits, do not actually claim them (Department for Education, 2012).

Limitations relating to CSC exposure measurement include that we did not examine CSC exposure in years 10/11. This was because we only planned to examine prospective associations as being referred to CSC services may be a consequence of leaving education early. While our analyses rule out this reverse causation, future research could be conducted into possible bi-directional associations between CSC exposure and non-enrolment. We also did not examine CSC exposure prior to year 4 (age 8/9 years) due to data not being collected before this point. As such, there were children misclassified as unexposed. As children with CSC history were more likely to experience non-enrolment, the effect of this misclassification would have been to reduce the association between CSC history and non-enrolment.

Strengths of the study include a prospective cohort using whole-population data that captured all children attending state schools. The measurement of CSC exposure prior to off-rolling in secondary school further means that our findings will be relevant to stakeholders, such as the Department for Education, Ofsted (the body responsible for inspecting schools) and schools themselves, when considering policy and interventions to retain these vulnerable pupils in education from age 11 until age 18 years (year 13).

### 4.3 Implications

#### 4.3.1 Policy implications

Our results relate to the large proportion of children who ever need CSC services in England before their 16th birthday (estimated to be at least 25%: Jay et al., 2020) and the 41% of children who receive SEND provision (Jay and Gilbert, 2021). Our methods can be routinely reproduced to monitor non-enrolment, and thereby potential off-rolling, in individual schools, academy chains, and local authorities. A priority should be to generate more certainty about when non-enrolment reflects off-rolling or disengagement from school to ensure prompt action and put in place adequate support for affected children. Such information could be used to identify children missing from education (Office of the Children’s Commissioner, 2021) and in Ofsted school inspections. The inspection framework now requires investigation of off-rolling (Ofsted, 2021), and states that a school is likely to be judged as inadequate on the leadership and management domain if found to be engaging in off-rolling (Ofsted, 2021). Evidence of persistent off-rolling could lead to sanctions, such as financial penalties or disciplinary measures for school leaders, as called for by the Children's Commissioner (Office of the Children’s Commissioner, 2011), and underpin evidence for legal claims by pupils. More urgent, however, is to ensure that affected children receive the support they need to prevent off-rolling and disengagement in the first place and support them when they do become unenrolled from school.

Preventive strategies to reduce disengagement from school and pushing out/off-rolling need to address the policy context of marketization of education and competition between schools. As Thomson notes, “[t]here is not as yet a positive policy incentive to encourage all children and young people to remain in school, grounded in a commitment to a full secondary education as a universal entitlement and benefit” (P. Thomson, 2020). Pushing out/off-rolling is driven by systematic factors meaning that schools are incentivized not to meet the complex needs of the most disadvantaged children—those in contact with CSC and those with SEND. This is true whether schools are acting deliberately to push out targeted children or whether they represent exclusionary environments unsuitable to children of diverse needs (Bonell et al., 2019). Necessary interventions are legal and regulatory and must be accompanied by sufficient resources to enable schools to adequately fulfill their duty to inclusively educate all children regardless of background. As the House of Commons Education Select Committee stated in its 2018 report on alternative provision and school exclusions, “[o]ff-rolling is created by the Department for Education. The Department cannot wash its hands of the issue, just as schools cannot wash their hands of their pupils” (House of Commons Education Committee, 2018). The right to education is a fundamental human right and the state, through CSC departments and schools, has a duty to protect it, particularly for the most vulnerable.

The implications for children who do not complete secondary schooling, whether this be due to off-rolling/pushing out, or a more general exclusionary environment created by national or local policies, are likely to be significant. Education is not only undoubtedly valuable for its own sake but, as noted in the introduction, higher levels of education are associated with a range of positive health and social outcomes across the life course. Education gives access to employment and social opportunities that are crucial for living a healthy and happy life. The immediate impacts of being pushed out/off-rolled, such as on academic attainment and propensity to be subject to gang involvement, must also be considered. Research should be conducted to understand the mechanisms of disengagement, non-enrolment and pushing out/off-rolling as well as these detrimental effects.

#### 4.3.2 Implications for research

Mixed-methods studies should investigate the mechanisms at school level that result in non-enrolment so that interventions can be tailored to monitor and address this problem. Studies should also address geographical variation in non-enrolment and how school and area-level factors, such as behavior policies and CSC organization and management, drive differential outcomes as well as the immediate and long-term consequences of becoming unenrolled from school. Cross-country comparisons of legal and regulatory frameworks, data availability and, where possible, non-enrolment and pushing out/off-rolling rates may elucidate possible interventions to protect the right to education of these vulnerable young people. Finally, all jurisdictions should consider what data would improve detection of pushing out using administrative data. In England, for example, private schools could contribute to the NPD in the same way as state schools and a national register of children in home school settings (Department for Education, 2019a), linkable to the NPD, could be established. Additionally, schools could be required to record reasons why children are removed from their rolls which, although pushing out/off-rolling would not be recorded, other reasons such as transfer to private school, migration, or death, could be ruled out in statistical analyses.

### 4.4 Conclusion

Children who need CSC services, and especially those with SEND, were at significantly heightened risk of becoming unenrolled from English state schools. These findings imply systemic, discriminatory breaches of the right to education of these specific groups of children. Reform is required to create a truly inclusive school system where all children, regardless of their background, are given the opportunity to flourish.

The following are the supplementary data related to this article.

Supplementary File 1 Analysis of first school year cohort inception. Supplementary File 1

Supplementary File 2 Social care definitions, cohort derivation and data cleaning. Supplementary File 2

Supplementary File 3 Characteristics of children with and without missing data. Supplementary File 3

Supplementary File 4 Cohort characteristics. Supplementary File 4

Supplementary File 5 Annual and cumulative proportions of non-enrolment by all variables. Supplementary File 5

Supplementary File 6 Full modelling results. Supplementary File 6

Supplementary File 7 Children in special schools. Supplementary File 7

## Data statement

Data used in this study cannot be shared by the authors. Researchers can apply to the Department for Education to access the data following the Department's approvals process (https://www.gov.uk/guidance/how-to-access-department-for-education-dfe-data-extracts, accessed 12 August 2021).

## Author contributions

MJ led on study design, to which all authors contributed. MJ carried out all data management and analyses. MJ, LMcGL and RG conducted the application to the Department for Education for access to the data. MJ drafted this manuscript to which all authors critically contributed.

## Declaration of competing interest

None.
