# Supplementary material for: Evaluation of pushing out of children from all English state schools: Administrative data cohort study of children receiving social care and their peers
Source: Child Abuse Negl. 2022 May;127:105582. doi: 10.1016/j.chiabu.2022.105582 (PMC9077441; doi:10.1016/j.chiabu.2022.105582)
Supplement: Supplementary File 1 — Analysis of first school year cohort inception. [file mmc1.docx]

# Supplementary File 1: Analysis of first school year cohort inception

In this Supplementary File is reported an analysis of a cohort defined by enrolment in the first year of school, rather than year 7. Within this Supplementary File, all numbers are rounded to the nearest 10 and all percentages <0.5% are suppressed; this was to comply with statistical disclosure rules as then in force when this file was exported from the Office for National Statistics Secure Research Service.

Two cohorts were defined:

- Cohort 1: must be enrolled in year 1 (aged 5/6 years) in 2005/6, n = 547,670. Of these, 63,600 (12%) had at least one year not enrolled.
- Cohort 2: must be enrolled in reception (aged 4/5 years) in 2005/6, n = 525,510. Of these, 66,790 (13%) had at least one year not enrolled.

Table S1.1 shows the number of rows loaded for cohort 1 when the 2005/6 file was loaded and then the number of rows after subsetting and adding children in alternative provision or pupil referral units. The final column gives the unique number of children. There were 547,670 children in this cohort.

Table S1.1. Number of rows and children in each census file for cohort 1

| File  AY (SY) | N rows on load | N rows after subsetting | N rows after addition of AP and PRU | N unique children |
| --- | --- | --- | --- | --- |
| *Initial file* |  |  |  |  |
| 2005/06 (1) | 7,625,670 | 547,740 | 547,740 | 547,670 |
|  |  |  |  |  |
| *Other years* |  |  |  |  |
| 2006/07 (2) | 7,564,710 | 539,240 | 539,240 | 539,130 |
| 2007/08 (3) | 7,513,170 | 531,500 | 531,680 | 531,520 |
| 2008/09 (4) | 7,483,990 | 526,280 | 526,530 | 526,400 |
| 2009/10 (5) | 7,504,520 | 522,640 | 523,200 | 523,000 |
| 2010/11 (6) | 7,532,200 | 519,730 | 520,570 | 520,340 |
| 2011/12 (7) | 7,585,890 | 508,880 | 510,410 | 509,740 |
| 2012/13 (8) | 7,655,720 | 506,580 | 508,900 | 508,100 |
| 2013/14 (9) | 7,751,150 | 505,390 | 507,200 | 506,620 |
| 2014/15 (10) | 7,854,020 | 503,080 | 505,660 | 504,850 |
| 2015/16 (11) | 7,974,950 | 497,450 | 501,140 | 500,400 |
|  |  |  |  |  |

AP alternative provision; AY academic year; PRU pupil referral unit; SY school year. One row is one enrolment (children may have multiple enrolments). The number of children in the “Other years” files after subsetting is after subsetting to the 547,670 children in the year 1 file. AP census started in 2007/08. The PRU census started in 2009/10 and was merged with the NPD census from 2013/14.

The analogous data for cohort 2 are shown in Table S1.2. There were 525,510 children in cohort 2.

Table S1.2. Number of rows and children in each census file for cohort 2

| File (school year) | N rows on load | N rows after subsetting | N rows after addition of AP and PRU | N unique children |
| --- | --- | --- | --- | --- |
| *Initial file* |  |  |  |  |
| 2005/06 (Reception) | 7,625,670 | 525,670 | 525,660 | 525,510 |
|  |  |  |  |  |
| *Other years* |  |  |  |  |
| 2006/07 (1) | 7,564,710 | 517,220 | 517,220 | 517,150 |
| 2007/08 (2) | 7,513,170 | 511,420 | 511,540 | 511,420 |
| 2008/09 (3) | 7,483,990 | 505,360 | 505,530 | 505,420 |
| 2009/10 (4) | 7,504,520 | 502,050 | 502,450 | 502,270 |
| 2010/11 (5) | 7,532,200 | 498,590 | 499,130 | 498,970 |
| 2011/12 (6) | 7,585,890 | 496,040 | 496,860 | 496,620 |
| 2012/13 (7) | 7,655,720 | 485,790 | 487,220 | 486,570 |
| 2013/14 (8) | 7,751,150 | 484,680 | 485,930 | 485,470 |
| 2014/15 (9) | 7,854,020 | 482,710 | 484,480 | 483,880 |
| 2015/16 (10) | 7,974,950 | 479,690 | 482,200 | 481,460 |
| 2016/17 (11) | 8,084,490 | 473,060 | 476,590 | 476,010 |
|  |  |  |  |  |

AP alternative provision; AY academic year; PRU pupil referral unit; SY school year. One row is one enrolment (children may have multiple enrolments). The number of children in the “Other years” files after subsetting is after subsetting to the 525,510 children in the reception year file. AP census started in 2007/08. The PRU census started in 2009/10 and was merged with the NPD census from 2013/14.

Table S1.3 shows that, among children who are ever not enrolled, the first year in which they become non-enrolled is year 7 for a substantial proportion. This is consistent with the hypothesis that these are children who attend state school for their primary education and then transfer to private education for their secondary. This was in fact the second most common enrolment pattern among the cohorts (Tables S1.4 and S1.5).

Table S1.3. Of all children ever not enrolled at least once, the first year in which children were not enrolled

| Year | Cohort 1 | Cohort 2 |
| --- | --- | --- |
|  | n (%) | n (%) |
|  |  |  |
| Reception | - | Inception point 2005/06 |
| 1 | Inception point 2005/06 | 8,360 (13%) |
| 2 | 8,530 (13%) | 6,910 (10%) |
| 3 | 8,770 (14%) | 7,480 (11%) |
| 4 | 6,580 (10%) | 4,850 (7%) |
| 5 | 5,240 (8%) | 4,790 (7%) |
| 6 | 4,240 (7%) | 3,830 (6%) |
| 7 | 13,520 (22%) | 12,750 (19%) |
| 8 | 3,320 (5%) | 3,020 (5%) |
| 9 | 3,730 (6%) | 3,610 (5%) |
| 10 | 3,850 (6%) | 4,330 (6%) |
| 11 | 5,820 (9%) | 6,870 (10%) |
|  |  |  |

Table S1.4. Top 20 enrolment patters in cohort 1

| Pattern No | n | % | y01 | y02 | y03 | y04 | y05 | y06 | y07 | y08 | y09 | y10 | y11 |
| --- | --- | --- | --- | --- | --- | --- | --- | --- | --- | --- | --- | --- | --- |
| 1 | 484,070 | 88.0 | 1 | 1 | 1 | 1 | 1 | 1 | 1 | 1 | 1 | 1 | 1 |
| 2 | 11,100 | 2.0 | 1 | 1 | 1 | 1 | 1 | 1 | 0 | 0 | 0 | 0 | 0 |
| 3 | 5,820 | 1.0 | 1 | 1 | 1 | 1 | 1 | 1 | 1 | 1 | 1 | 1 | 0 |
| 4 | 5,480 | 1.0 | 1 | 1 | 0 | 0 | 0 | 0 | 0 | 0 | 0 | 0 | 0 |
| 5 | 5,150 | 0.9 | 1 | 0 | 0 | 0 | 0 | 0 | 0 | 0 | 0 | 0 | 0 |
| 6 | 4,020 | 0.7 | 1 | 1 | 1 | 0 | 0 | 0 | 0 | 0 | 0 | 0 | 0 |
| 7 | 3,280 | 0.6 | 1 | 1 | 1 | 1 | 0 | 0 | 0 | 0 | 0 | 0 | 0 |
| 8 | 2,830 | 0.5 | 1 | 1 | 1 | 1 | 1 | 1 | 1 | 1 | 1 | 0 | 0 |
| 9 | 2,740 | 0.5 | 1 | 1 | 1 | 1 | 1 | 0 | 0 | 0 | 0 | 0 | 0 |
| 10 | 2,450 | 0.5 | 1 | 1 | 1 | 1 | 1 | 1 | 1 | 1 | 0 | 0 | 0 |
| 11 | 2,010 | <0.5 | 1 | 1 | 1 | 1 | 1 | 1 | 1 | 0 | 0 | 0 | 0 |
| 12 | 1,020 | <0.5 | 1 | 1 | 1 | 1 | 1 | 1 | 1 | 1 | 1 | 0 | 1 |
| 13 | 940 | <0.5 | 1 | 1 | 1 | 1 | 1 | 1 | 1 | 1 | 0 | 1 | 1 |
| 14 | 910 | <0.5 | 1 | 1 | 1 | 1 | 1 | 1 | 0 | 1 | 1 | 1 | 1 |
| 15 | 790 | <0.5 | 1 | 0 | 1 | 1 | 1 | 1 | 1 | 1 | 1 | 1 | 1 |
| 16 | 780 | <0.5 | 1 | 1 | 1 | 1 | 1 | 1 | 1 | 0 | 1 | 1 | 1 |
| 17 | 750 | <0.5 | 1 | 1 | 0 | 1 | 1 | 1 | 1 | 1 | 1 | 1 | 1 |
| 18 | 740 | <0.5 | 1 | 1 | 1 | 0 | 1 | 1 | 1 | 1 | 1 | 1 | 1 |
| 19 | 690 | <0.5 | 1 | 1 | 1 | 1 | 1 | 1 | 0 | 0 | 1 | 1 | 1 |
| 20 | 670 | <0.5 | 1 | 1 | 0 | 0 | 0 | 0 | 1 | 1 | 1 | 1 | 1 |
| All Others | 35,330 | 6.0 | Various patterns | | | | | | | | | | |

1 = enrolled in state school that year; 0 = not enrolled. All children were enrolled in year 1 by virtue of this being the cohort inception point.

Table S1.5. Top 20 enrolment patters in cohort 2

| Pattern No | n | % | y00 | y01 | y02 | y03 | y04 | y05 | y06 | y07 | y08 | y09 | y10 | y11 |
| --- | --- | --- | --- | --- | --- | --- | --- | --- | --- | --- | --- | --- | --- | --- |
| 1 | 458,720 | 87.0 | 1 | 1 | 1 | 1 | 1 | 1 | 1 | 1 | 1 | 1 | 1 | 1 |
| 2 | 10,420 | 2.0 | 1 | 1 | 1 | 1 | 1 | 1 | 1 | 0 | 0 | 0 | 0 | 0 |
| 3 | 6,870 | 1.0 | 1 | 1 | 1 | 1 | 1 | 1 | 1 | 1 | 1 | 1 | 1 | 0 |
| 4 | 5,010 | 1.0 | 1 | 0 | 0 | 0 | 0 | 0 | 0 | 0 | 0 | 0 | 0 | 0 |
| 5 | 4,670 | 0.9 | 1 | 1 | 1 | 0 | 0 | 0 | 0 | 0 | 0 | 0 | 0 | 0 |
| 6 | 4,010 | 0.8 | 1 | 1 | 0 | 0 | 0 | 0 | 0 | 0 | 0 | 0 | 0 | 0 |
| 7 | 3,260 | 0.6 | 1 | 1 | 1 | 1 | 1 | 1 | 1 | 1 | 1 | 1 | 0 | 0 |
| 8 | 2,960 | 0.6 | 1 | 1 | 1 | 1 | 1 | 0 | 0 | 0 | 0 | 0 | 0 | 0 |
| 9 | 2,870 | 0.6 | 1 | 1 | 1 | 1 | 0 | 0 | 0 | 0 | 0 | 0 | 0 | 0 |
| 10 | 2,550 | 0.5 | 1 | 1 | 1 | 1 | 1 | 1 | 0 | 0 | 0 | 0 | 0 | 0 |
| 11 | 2,410 | 0.5 | 1 | 1 | 1 | 1 | 1 | 1 | 1 | 1 | 1 | 0 | 0 | 0 |
| 12 | 1,850 | <0.5 | 1 | 1 | 1 | 1 | 1 | 1 | 1 | 1 | 0 | 0 | 0 | 0 |
| 13 | 1,070 | <0.5 | 1 | 1 | 1 | 1 | 1 | 1 | 1 | 1 | 1 | 1 | 0 | 1 |
| 14 | 980 | <0.5 | 1 | 1 | 1 | 1 | 1 | 1 | 1 | 0 | 1 | 1 | 1 | 1 |
| 15 | 880 | <0.5 | 1 | 1 | 1 | 1 | 1 | 1 | 1 | 1 | 1 | 0 | 1 | 1 |
| 16 | 790 | <0.5 | 1 | 0 | 1 | 1 | 1 | 1 | 1 | 1 | 1 | 1 | 1 | 1 |
| 17 | 720 | <0.5 | 1 | 1 | 0 | 1 | 1 | 1 | 1 | 1 | 1 | 1 | 1 | 1 |
| 18 | 670 | <0.5 | 1 | 1 | 1 | 1 | 1 | 1 | 1 | 1 | 0 | 1 | 1 | 1 |
| 19 | 670 | <0.5 | 1 | 1 | 1 | 0 | 1 | 1 | 1 | 1 | 1 | 1 | 1 | 1 |
| 20 | 600 | <0.5 | 1 | 1 | 1 | 1 | 1 | 1 | 1 | 0 | 0 | 1 | 1 | 1 |
| All Others | 27,840 | 5.0 | Various patterns | | | | | | | | | | | |

1 = enrolled in state school that year; 0 = not enrolled. All children were enrolled in year 1 by virtue of this being the cohort inception point.
