# Supplementary material for: Evaluation of pushing out of children from all English state schools: Administrative data cohort study of children receiving social care and their peers
Source: Child Abuse Negl. 2022 May;127:105582. doi: 10.1016/j.chiabu.2022.105582 (PMC9077441; doi:10.1016/j.chiabu.2022.105582)
Supplement: Supplementary File 2 — Social care definitions, cohort derivation and data cleaning. [file mmc2.docx]

## Supplementary File 2: social care definitions, cohort derivation and data cleaning

## S2.1. Introduction

In this Supplementary File are detailed the process by which we derived the cohort and the steps undertaken to ensure the data were analysis ready. It is divided into eight sections: this introduction (S2.1); social care definitions (S2.2); ethics and data protection (S2.3); National Pupil Database (NPD) preparation (S2.4); children looked after (CLA) data preparation (S2.5); children in need (CiN) data preparation (S2.6); education – social care linkage (S2.7); references in this Supplementary File (S2.8). Throughout this Supplementary File, cohort 1 are the children enrolled in year 7 in 2011/12 (or aged 11 at the start of that academic year if not following the National Curriculum) and cohort 2 are those in year 7 in 2012/13 (or aged 11 at the start of that academic year if not following the National Curriculum). Citations are unique to this Supplementary File and are given at the end of this document.

Supplementary File 2: social care definitions, cohort derivation and data cleaning 1

S2.1. Introduction 1

S2.2. Social care definitions 2

S2.3. Ethics and data protection 4

S2.4. National Pupil Database (NPD) preparation 5

Data cleaning 7

Addition of school-level data 8

S2.5. Children looked after (CLA) data preparation 9

S2.6. Children in Need (CiN) data preparation 12

S2.7. Education – social care linkage 15

S2.8. References in this Supplementary File 17

## S2.2. Social care definitions

Three children’s social care (CSC) exposure categories were identified in the study: children in need (CiN), children subject to child protection plans (CPPs) and children looked after (CLA).

#### Children in need

In England, local authorities (LAs) have duties and powers for the provision of services for CiN. A child is in need, per section 17(10) of the Children Act 1989, if:

(a) [they are] unlikely to achieve or maintain, or to have the opportunity of achieving or maintaining, a reasonable standard of health or development without the provision for [them] of services by a local authority…;

(b) [their] health or development is likely to be significantly impaired, or further impaired, without the provision for [them] of such services; or

(c) [they are] disabled.

CiN are children therefore who require additional help from the LA to support or maintain their health and well-being and/or who are disabled. The population of CiN is large. Each year, about 5% of children are referred to CSC and about 3% of all children are CiN (Department for Education, 2021a). Of all children born in England, 43% are estimated to be referred to CSC before they turn 16 and 25% are found to be CiN (Jay et al., 2020).

#### Child protection plans

A sub-group of CiN are children on CPPs. A CPP is a possible outcome of LA child protection investigations, i.e., where a child is known or suspected to be suffering or at risk of suffering significant harm. The purpose of a CPP is to ensure the child is kept safe, to promote the child’s health and development and to support the family and wider family to safeguard and promote the welfare of the child (Department for Education, 2020b). A plan might include, for example, regular social work visits or home help. A plan will usually make clear that if parents do not comply, the LA will instigate care proceedings. Therefore, the imposition of a CPP can be seen as a level of child protection intervention above services for CiN (which may or may not relate to child protection concerns) but below provision for CLA (which, likewise, may or may not be in response to child protection concerns).

#### Children looked after

CLA can be considered in two broad groups: those who enter care compulsorily, mostly by court order, and those are accommodated by a LA. In this study, both groups are treated the same.

#### Entering care

A court may make a care order under section 31 of the Children Act 1989 where it is satisfied that the child is suffering or likely to suffer significant harm and that the harm is attributable to ‘the care given to the child not being what it would be reasonable to expect a parent to give’ or to the child’s being beyond parental control. The LA can also apply for an interim care order or emergency protection order, and the police may remove a child to a place of safety in an emergency. Finally, children can enter care by being detained under criminal justice provisions. All children who enter care via these routes are CLA.

#### Being accommodated

A child may also become a CLA via the extra-judicial route under section 20 of the Children Act 1989. This is available in situations where there are no parents, such as unaccompanied asylum-seeking children or orphaned children, or where the parents are unable or unwilling to provide accommodation and the LA’s accommodating the child would promote the child’s welfare.

Regardless of legal status, approximately 0.65% of the childhood population are looked after each year (Department for Education, 2021b). Of children born between 1992 and 1994, 3.3% were looked after at least once before their 18^th^ birthday and this cumulative incidence appears to be rising birth cohorts since the turn of the millennium (Mc Grath-Lone et al., 2015).

## S2.3. Ethics and data protection

Initially, the chair of the University College London ethics committee confirmed that ethical approval was not required as we were working with anonymized data. We registered this project with University College London’s research and development office (17PE25) and with our data protection officer (Z6364106 2020 07 51). Following a change in University College London’s policy relating to data from vulnerable populations, including all children, we applied for and received ethical approval from the University College London research ethics committee (11483/001).

Data were shared by the Department for Education under a legally binding data sharing agreement and were initially stored in the University College London data safe haven. In March 2020, data were transferred to the Office for National Statistics Secure Research Service (Department for Education, 2020a) as per new Department for Education requirements.

All outputs from the University College London safe haven were independently checked by a data scientist and all outputs from the Secure Research Service were checked at least twice by Office for National Statistics staff to ensure compliance with statistical disclosure controls. These controls included a threshold of 10, below which cell counts could not be reported.

## S2.4. National Pupil Database (NPD) preparation

A flow diagram representing the process by which we derived the cohort is given in Figure S2.1.

Figure S2.1. Flow diagram summarizing dataset production


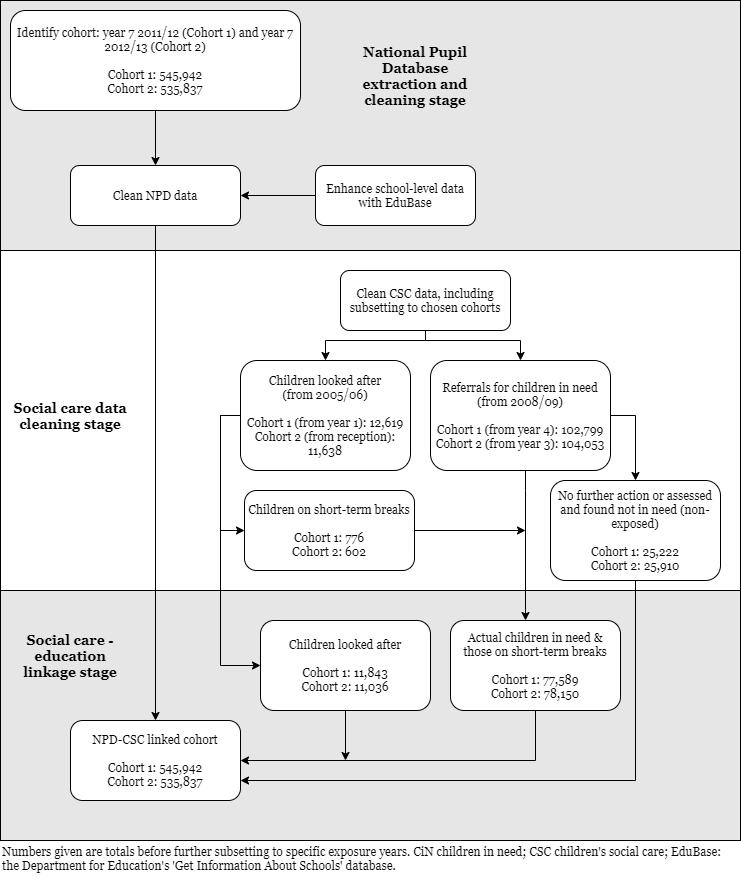


Each NPD spring census is provided by the Department for Education (DfE) as a separate file containing all children enrolled in English state schools, where each row represents one enrolment (approximately 7.6 million per census year). For cohort 1, the cohort inception file was the 2011/12 file, for cohort 2, the 2012/13 file.

To identify cohort 1, we first loaded the 2011/12 file (7,585,888 rows) and we subsetted to all children who were in year 7 or, where children were not following the National Curriculum, who were 11 years old at the start of the academic year. This resulted in 544,925 rows. We then added enrolments in 2011/12 in the alternative provision (AP) and PRU censuses, resulting in 546,646 rows. These were of 545,942 unique children (compared to 544,835 in official DfE statistics on state school enrolments (Department for Education, 2012). In England, there were 586,219 children aged 11 in 2012 (Office for National Statistics, 2021). Cohort 1 was therefore approximately 93% of the population, the difference mostly accountable by pupils in the independent sector or who are educated at home.

Next, we loaded the other census files and subsetted each to the children in the cohort. Where available, we added children in the AP and PRU censuses. The numbers at each stage are given in Table S2.1.

Table S2.1. Number of rows and children in each census file for cohort 1

| File  AY (SY) | N rows on load* | N rows after subsetting* | N rows after addition of AP and PRU† | N unique children |
| --- | --- | --- | --- | --- |
| *Initial file* |  |  |  |  |
| 2011/12 (7) | 7,585,888 | 544,925 | 546,646 | 545,942 |
|  |  |  |  |  |
| *Other years* |  |  |  |  |
| 2005/06 (1) | 7,625,666 | 508,927 | 508,927 | 508,858 |
| 2006/07 (2) | 7,564,710 | 513,953 | 513,953 | 513,873 |
| 2007/08 (3) | 7,513,168 | 518,192 | 518,459 | 518,282 |
| 2008/09 (4) | 7,483,985 | 522,318 | 522,635 | 522,497 |
| 2009/10 (5) | 7,504,521 | 527,015 | 527,643 | 527,429 |
| 2010/11 (6) | 7,532,199 | 532,213 | 533,164 | 532,918 |
| 2012/13 (8) | 7,655,721 | 538,998 | 541,410 | 540,552 |
| 2013/14 (9) | 7,751,148 | 535,194 | 537,101 | 536,445 |
| 2014/15 (10) | 7,854,018 | 531,378 | 534,025 | 533,160 |
| 2015/16 (11) | 7,974,945 | 525,290 | 529,066 | 528,278 |

AP alternative provision; AY academic year; PRU pupil referral unit; SY school year. * One row is one enrolment (children may have multiple enrolments). The number of children in the “Other years” files after subsetting is after subsetting to the 544,925 children in the year 7 file. † AP census started in 2007/08. The PRU census started in 2009/10 and was merged with the NPD census from 2013/14.

Once all files were loaded, we merged them into one long-format file where each row represents one enrolment in a state school and where a child can have more than one enrolment per year. We created flags to indicate dually-enrolled so they were not double counted.

The same process was repeated with cohort 2, starting with the 2012/13 spring census as the inception file. The numbers at each stage are given in Table S2.2. There was a total of 535,837 unique children in cohort 2. In both cohorts, therefore, there were a total of 1,081,779 children.

Table S2.2. Number of rows and children in each census file for cohort 2

| File  AY (SY) | N rows on load* | N rows after subsetting* | N rows after addition of AP and PRU† | N unique children |
| --- | --- | --- | --- | --- |
| *Initial file* |  |  |  |  |
| 2011/12 (7) | 7,655,721 | 535,051 | 536,545 | 535,837 |
|  |  |  |  |  |
| *Other years* |  |  |  |  |
| 2005/06 (R) | 7,625,666 | 491,275 | 491,275 | 491,105 |
| 2006/07 (1) | 7,564,710 | 500,276 | 500,276 | 500,209 |
| 2007/08 (2) | 7,513,168 | 504,678 | 504,855 | 504,736 |
| 2008/09 (3) | 7,483,985 | 508,680 | 508,912 | 508,790 |
| 2009/10 (4) | 7,504,521 | 512,794 | 513,287 | 513,077 |
| 2010/11 (5) | 7,532,199 | 517,296 | 517,942 | 517,756 |
| 2012/13 (6) | 7,585,888 | 522,556 | 523,501 | 532,241 |
| 2013/14 (8) | 7,751,148 | 529,810 | 531,146 | 530,625 |
| 2014/15 (9) | 7,854,018 | 525,191 | 527,034 | 526,394 |
| 2015/16 (10) | 7,974,945 | 520,485 | 523,113 | 522,337 |
| 2016/17 (11) | 8,084,492 | 513,472 | 517,137 | 516,513 |

AP alternative provision; AY academic year; PRU pupil referral unit; R reception year; SY school year. * One row is one enrolment (children may have multiple enrolments). The number of children in the “Other years” files after subsetting is after subsetting to the 544,925 children in the year 7 file. † AP census started in 2007/08. The PRU census started in 2009/10 and was merged with the NPD census from 2013/14.

### Data cleaning

We checked for inconsistent gender, ethnicity and first language values (ignoring missing values) within children’s records across time, including across the spring census files and the AP and PRU censuses, where available. Table S2.3 shows the number and proportion of children in each cohort, and overall, who had inconsistencies.

Table S2.3. Number and proportion of children with inconsistent characteristics data across enrolments

| Variable | Cohort 1  (n = 545,942) | Cohort 2  (n = 535,837) | Total  (n = 1,081,779) |
| --- | --- | --- | --- |
|  |  |  |  |
| Gender | 3314 (0.6%) | 4421 (0.8%) | 7735 (0.7%) |
| Ethnicity | 62545 (11.5%) | 72985 (13.6%) | 135,530 (12.5%) |
| First language | 42784 (7.8%) | 70943 (13.2%) | 113,727 (10.5%) |
|  |  |  |  |

To resolve inconsistencies, the modal value was taken from across all that child’s records, missing values ignored. In cases of multimodal values, one of the modes was chosen at random.

### Addition of school-level data

We imported school-level data from the “Get information about schools” database (Department for Education, 2018), using the school Unique Reference Number to link data. Except for AP establishments, all schools appearing among the cohorts’ schools were also in EduBase, meaning there was a 100% match rate.

## S2.5. Children looked after (CLA) data preparation

Two CLA files were provided by DfE: an episodes file and a children file. The children file contained the last episode in each academic year whereas the episodes file contained all episodes. The children file was therefore redundant except that only it contained the Pupil Matching Reference (PMR). The PMR therefore had to be imported into the episodes file using the encrypted local authority (LA) child identifier.

Cleaning steps are detailed in Table S2.4. The final CLA dataset consisted of 86,480 episodes from 22,879 unique children across primary (age 4 to 11 years) and secondary school (age 11 to 16 years) for both cohorts (cohort 1: 11,843; cohort 2: 11,036). There were additionally 34,764 episodes from 2,039 children on short-term breaks over the same period.

Table S2.4. Children looked after (CLA) data cleaning

|  | **Cleaning step** |  | **Number after cleaning**  Rows (Children) |
| --- | --- | --- | --- |
| 1 | Load whole CLA dataset (includes children looked after from April 2005). |  | E: 1,468,377 (329,226)  Ch: 982,159 (266,547) |
|  |  |  |  |
| 2 | LA-child identifier number duplicated across LAs. Different LAs use their own numbering systems and different children in different LAs might inadvertently be assigned the same number. This step completed on the children file. | 0 found. |  |
|  |  |  |  |
| 3 | Fill back PMRs within same child. Where a child enters care before school but is later assigned a PMR, their earlier records will be missing their PMR. This step completed on the children file. | 318 rows filled in. |  |
|  |  |  |  |
| 4 | Import PMR into episodes data file. Drop the children file. All subsequent cleaning steps completed on the episodes file. |  |  |
|  |  |  |  |
| 5 | Drop children not in cohort 1 (i.e., not enrolled in state school in year 7 in 2011/12 or aged 11 at the start of that academic year [C1] or year 7 in 2012/13 or aged 11 [C2]). | A large proportion of rows were dropped because they were of children of all ages and in other school cohorts. | C1: 65,250 (12,564)  C2: 61,049 (11,617) |
|  |  |  |  |
| 6 | PMRs shared among multiple LA child IDs (see text, below, for rationale). | 3,240 (C1) / 2,820 (C2) episodes of children where a PMR was matched to multiple child IDs. See text, below, for details. | C1: 65,219 (12,559)  C2: 61,022 (11,613) |
|  |  |  |  |
| 7 | Dates:   - Missing episode start and end dates and period of care start dates. - Episode end date < episode start date. - Episode start date < period of care start date. - Period of care start date < date of birth. | 1 row dropped. | C1: 65,218 (12,559)  C2: 61,022 (11,613) |
|  |  |  |  |
| 8 | Removal of duplicates based on coincidence of LA child ID, period of care start date, episode start date, placement type and legal status. |  | C1: 65,216 (12,559)  C2: 61,016 (11,613) |
|  |  |  |  |
| 9 | Drop short-term break episodes. | The file for children on short-term breaks (C1: 17,431 episodes of 1,110 children; C2: 17,333 episodes of 929 children) was retained for later use. | C1: 47,785 (11,988)  C2: 43,683 (11,106) |
|  |  |  |  |
| 10 | Check missing episode end dates. | There was a total of 4,612 missing episode end dates. These were of episodes in the last year where a missing end date is valid and so no action was taken. |  |
|  |  |  |  |
| 11 | Drop episodes ending < 01/09/2005 and children with > 100 episodes (deemed to be in error in the legal status; merged with this step to avoid small cell counts). |  | C1: 45,379 (11,843)  C2: 41,101 (11,036) |
|  |  |  |  |
| 12 | Start date of first episode in period of care = period of care start date. | There were mismatches in: C1: 391 episodes; C2: 297 episodes. All period of care start dates set to first episode start date. |  |
|  |  |  |  |
| 13 | Cleaned dataset. |  | C1: 45,379 (11,843)  C2: 41,101 (11,036) |
|  |  |  |  |

C children file; C1 cohort 1; C2 cohort 2; E episodes file; LA local authority; NPD National Pupil Database; PMR Pupil Matching Reference.

Step 6 of Table S2.4 refers to an algorithm which cleaned PMRs that were shared across multiple LA-child identifiers (an identifier code that the LA assigns locally). This would occur in one of two situations. First, if a child is looked after by different LAs, the child will retain their PMR, which is nationally unique, but will be assigned a new LA-child identifier because each LA uses its own numbering system. Alternatively, two different children may have been assigned the same PMR in error (i.e., linkage error). To minimize the risk of linkage error, we created a rule-based algorithm that determined that where a PMR was shared among multiple LA child identifiers, those different LA child identifiers were deemed to truly belong to the same child if any two of the date of birth, gender or ethnicity were the same. Therefore, where two or more of these variables were different, the PMR was deemed to have been assigned to different children in error and these children were dropped. There were 3,240 episodes in cohort 1 (of all 65,250, 5%) where a PMR was shared among multiple LA child identifiers. In cohort 2, there were 2,820 of 61,049 (5%). Of all affected episodes, there were just 58 rows (<10 children) where at least two of date of birth, gender or ethnicity clashed. These children were dropped.

## S2.6. Children in Need (CiN) data preparation

On loading the CiN data file, there were 6,945,317 rows and 1,919,643 children. The cleaning rules as detailed in Table S2.5 were applied.

Table S2.5. Children in need census cleaning

|  | **Cleaning step** |  | **Number after cleaning**  Rows (Children) |
| --- | --- | --- | --- |
| 1 | Load whole CiN dataset (includes all children referred to children’s social care since October 2008). |  | 6,945,317 (1,919,643) |
|  |  |  |  |
| 2 | Drop children not in cohort 1 or 2 (i.e., not enrolled in state school in year 7 in 2011/12 or aged 11 at the start of that academic year, or in year 7 in 2012/13 or aged 11 at the start of that academic year.  Remove episode finished < 01/04/2008. These are historical episodes and are incomplete (aggregated here to avoid small cell counts). | A large proportion of rows were dropped because they were of children of all ages and in other school cohorts. | C1: 342,421 (106,154)  C2: 337,673 (105,816) |
|  |  |  |  |
| 3 | PMRs shared among multiple LA-child IDs (see text, below, for rationale). | 63,130 (C1) / 62,400 (C2) rows of children where a PMR was matched to multiple child IDs. See text, below, for details. | C1: 342,129 (106,103)  C2: 337,443 (105,778) |
|  |  |  |  |
| 4 | Removal of duplicates based on coincidence of PMR, academic year, date of birth, gender, disability, local authority, referral date, closure date, primary need code, reason for closure, referral source, ethnicity, category of abuse, initial category of abuse, latest category of abuse, child protection plan start date and child protection plan end date. |  | C1: 342,097 (106,103)  C2: 337,399 (105,778) |
|  |  |  |  |
| 5 | Check consistency of end dates across episode. | There were no episodes with inconsistent end dates.* |  |
|  |  |  |  |
| 6 | Referral date > closure date (again). | 44 (C1) / 51 (C2) rows found. Closure date was set to referral on the assumption that the later record would be more accurate. |  |
|  |  |  |  |
| 7 | Second de-duplication on same variables at step 4.  Episode finished < 01/09/2008. These episodes were dropped to ensure homogeneity across all children in terms of being able to be observed in the dataset (aggregated here to avoid small cell counts).  Check date of birth > referral date (aggregated here to avoid small cell counts). |  | C1: 341,551 (106,050)  C2: 336,640 (105,716) |
|  |  |  |  |
| 9 | Referral source consistent across episode.* | 42,673 (C1) / 40,140 (C2) rows fixed.* |  |
|  |  |  |  |
| 10 | ‘No further action’ variable consistent across episode.* | 44,337 (C1) / 42,460 (C2) rows fixed.* |  |
|  |  |  |  |
| 11 | ‘No further action’ is missing. | 12,060 (C1) / 12,340 (C2) rows fixed. It was assumed that these were in fact Noes. |  |
|  |  |  |  |
| 12 | ‘Primary need code’ variable consistent across episode.* | 19,402 (C1) / 18,404 (C2) rows fixed.* |  |
|  |  |  |  |
| 13 | Closure reason consistent across episode.* | 112,553 (C1) / 108,377 (C2) rows fixed.* |  |
|  |  |  |  |
| 14 | Disability consistent across episode.* | 29,096 (C1) / 26,969 (C2) rows fixed.* |  |
|  |  |  |  |
| 15 | Ethnicity consistent across episode.* | 5,905 (C1) / 6,010 (C2) rows fixed.* |  |
|  |  |  |  |
| 16 | Gender consistent across episode.* | 417 (C1) / 338 (C2) rows fixed.* |  |
|  |  |  |  |
| 17 | Check for missing end dates of no further action referrals. | 18,243 (C1) / 17,715 (C2) missing end dates were set to the referral’s start date. |  |
|  |  |  |  |
| 19 | Check for inconsistent CPP start dates within episodes.* | 2,254 (C1) / 2,525 (C2) rows with inconsistent CPP start dates fixed.* |  |
|  |  |  |  |
| 20 | Final de-duplication on same variables at step 4. |  | C1: 341,403 (106,050)  C2: 336,516 (105,716) |
|  |  |  |  |
| 21 | Cleaned dataset. |  | C1: 341,403 (106,050)  C2: 336,516 (105,716) |
|  |  |  |  |

C1 cohort 1; C2 cohort 2; CiN children in need; CPP child protection plan; LA local authority; NPD National Pupil Database; PMR Pupil Matching Reference. * Because the LA can return the same episode more than once (which gives multiple rows per episode), there is potential for error being introduced with each return resulting in non-time-varying data apparently varying. For these variables, the modal value across each of the episode’s rows was taken or, where this was multimodal, the latest value was taken.

Step 3 of Table S2.5 refers to an algorithm which cleaned PMRs that were shared across multiple LA-child identifiers. We applied the same rules as with the CLA data (above). There were 63,126 (cohort 1) and 62,397 (cohort 2) rows in total where a PMR was shared among multiple LA-child identifiers. Of these affected episodes, there were 292 rows (51 children) in cohort 1, and 230 rows (38 children) in cohort 2, where at least two of date of birth, gender or ethnicity clashed. These children were dropped.

The cleaning of the CiN dataset resulted in a dataset of 677,919 rows of 211,766 unique children from year 3 or 4 (ages 6-8, depending on cohort) to year 11 (age 15/16). Within this, however, were children who were referred to CSC but not assessed or who were assessed but found not to be in need. The numbers of unique children referred, assessed and found to be CiN are given in Table S2.6. Because CiN data were available from year 4 for cohort 1 and year 3 for cohort 2, Table S2.6 is subset to years 4 to 6 in the primary phase for the sake of consistency in timing between the two cohorts.

Table S2.6. Number of unique children referred, assessed and found to be children in need

| School phase | Referred | Assessed | CiN |
| --- | --- | --- | --- |
|  |  |  |  |
| *Cohort 1 (n = 545,942)* |  |  |  |
| Primary (yr 4 to 6) | 54,524 (10%) | 50,144 (9%) | 40,985 (8%) |
| Secondary (yr 7 to 11) | 85,564 (16%) | 81,217 (15%) | 63,253 (12%) |
| Total (yr 4 to 11)* | 102,799 (19%) | 96,896 (18%) | 77,577 (14%) |
|  |  |  |  |
| *Cohort 2 (n = 535,837)* |  |  |  |
| Primary (yr 4 to 6) | 61,418 (11%) | 56,738 (11%) | 44,905 (8%) |
| Secondary (yr 7 to 11) | 81,956 (15%) | 77,968 (15%) | 61,520 (11%) |
| Total (yr 4 to 11)* | 104,053 (19%) | 98,126 (18%) | 78,143 (15%) |
|  |  |  |  |

The denominator for all percentages is the number of children in each cohort. * The numbers of referrals, etc., do not sum to the totals as children may be referred, etc., in both periods. CiN children in need.

## S2.7. Education – social care linkage

Finally, we linked the CLA and CiN datasets to the NPD using the PMR. Of the 1,081,779 children in the cohorts, there were, as indicated above, 22,879 (2.1%) who had been looked after during primary or secondary school.

Obtaining the number of children ever in need was slightly more complex because children who were on STBs (and who were therefore originally counted in the CLA dataset) were to be counted as CiN. As the CiN dataset began in the academic year 4 in 2008/09 for cohort 1, and year 3 in 2008/09 for cohort 2, the data are only available for the latter part of primary school for both cohorts. We therefore only counted CiN and STB episodes from year 4 in both cohorts. Table S2.7 gives the number of children found to be CiN (i.e., the final column in Table S2.6), the number of children on STBs and the final number of CiN and children on STBs.

Table S2.7. Children in need, children on short-term breaks and the two combined

| Cohort | CiN | STBs | CiN & STBs* |
| --- | --- | --- | --- |
|  |  |  |  |
| *Cohort 1 (n = 545,942)* |  |  |  |
| Primary (yr 4 to 6) | 40,985 (8%) | 495 (0.1%) | 41,002 (8%) |
| Secondary (yr 7 to 11) | 63,253 (12%) | 413 (0.1%) | 63,258 (12%) |
| Total (yr 4 to 11) | 77,577 (14%) | 776 (0.1%) | 77,589 (14%) |
|  |  |  |  |
| *Cohort 2 (n = 535,837)* |  |  |  |
| Primary (yr 4 to 6) | 44,905 (8%) | 408 (0.1%) | 44,920 (8%) |
| Secondary (yr 7 to 11) | 61,520 (11%) | 307 (0.1%) | 61,522 (11%) |
| Total (yr 4 to 11) | 78,143 (15%) | 602 (0.1%) | 78,150 (15%) |
|  |  |  |  |

* The number of actual CiN plus the number of those on STBs (final column) is not that much higher than the number who were found to be CiN (second column) because most children on STBs had been recorded at some point in the CiN census as well as the CLA dataset for their STBs episode. CiN children in need; STBs short-term breaks.

In total, there were therefore 155,739 (14%) children who were CiN or on STBs at any time from year 4 to 11 in both cohorts.

## S2.8. References in this Supplementary File

Department for Education. (2012). *Schools, pupils and their characteristics: January 2012*. https://www.gov.uk/government/statistics/schools-pupils-and-their-characteristics-january-2012

Department for Education. (2018). *Get information about schools*. https://get-information-schools.service.gov.uk/

Department for Education. (2020a). *How to access Department for Education (DfE) data extracts*. https://www.gov.uk/guidance/how-to-access-department-for-education-dfe-data-extracts

Department for Education. (2020b). *Working together to safeguard children*. https://www.gov.uk/government/publications/working-together-to-safeguard-children--2

Department for Education. (2021a). *Statistics: children in need and child protection*. https://www.gov.uk/government/collections/statistics-children-in-need

Department for Education. (2021b). *Statistics: looked-after children*. https://www.gov.uk/government/collections/statistics-looked-after-children

Jay, M. A., Stavola, B. De, Dorsett, R., Thomson, D., & Gilbert, R. (2020). *Model estimates of cumulative incidence of children in need status and referral to children’s social care (pre-print). https://osf.io/6ecrz/*. https://doi.org/10.17605/OSF.IO/6ECRZ

Mc Grath-Lone, L., Dearden, L., Nasim, B., Harron, K., & Gilbert, R. (2015). Changes in first entry to out-of-home care from 1992 to 2012 among children in England. *Child Abuse & Neglect*, *51*, 163–171. https://doi.org/10.1016/j.chiabu.2015.10.020

Office for National Statistics. (2021). *Estimates of the population for the UK, England and Wales, Scotland and Northern Ireland*. https://www.ons.gov.uk/peoplepopulationandcommunity/populationandmigration/populationestimates/datasets/populationestimatesforukenglandandwalesscotlandandnorthernireland
