# Supplementary material for: Evaluation of pushing out of children from all English state schools: Administrative data cohort study of children receiving social care and their peers
Source: Child Abuse Negl. 2022 May;127:105582. doi: 10.1016/j.chiabu.2022.105582 (PMC9077441; doi:10.1016/j.chiabu.2022.105582)
Supplement: Supplementary File 3 — Characteristics of children with and without missing data. [file mmc3.docx]

## Supplementary File 3: characteristics of children with and without missing data

Table S3.1. Characteristics of children with and without missing data (n = 1,081,799 children)

| Variable |  | Any missing | No missing |
| --- | --- | --- | --- |
|  |  | n (%) | n (%) |
| n |  | 5,469 (0.5%)^a^ | 1,076,310 (99.5%)^a^ |
| CSC exposure (yr 4 to 6) | None | 4,252 (77.7%) | 991,118 (92.1%) |
|  | CiN | 866 (15.8%) | 73,370 (6.8%) |
|  | CPP | 65 (1.2%) | 56,88 (0.5%) |
|  | CLA | 286 (5.2%) | 61,34 (0.6%) |
|  |  |  |  |
| Gender | Male | 3,343 (61.1%) | 550,760 (51.2%) |
|  | Female | * | * |
|  | Missing | * | * |
|  |  |  |  |
| Ethnicity | White | 3,303 (60.4%) | 856,318 (79.6%) |
|  | Black | 253 (4.6%) | 55,143 (5.1%) |
|  | Mixed | 221 (4.0%) | 45,069 (4.2%) |
|  | Asian | 239 (4.4%) | 104,519 (9.7%) |
|  | Other | 68 (1.2%) | 15,261 (1.4%) |
|  | Missing | 1,385 (25.3%) | N/A |
|  |  |  |  |
| Language | English | 4,625 (84.6%) | 906,326 (84.2%) |
|  | Other | 623 (11.4%) | 169,984 (15.8%) |
|  | Missing | 221 (4.0%) | N/A |
|  |  |  |  |
| IDACI fifths | 1 (most deprived) | 219 (4.0%) | 256,983 (23.9%) |
|  | 2 | 270 (4.9%) | 223,594 (20.8%) |
|  | 3 | 267 (4.9%) | 204,721 (19.0%) |
|  | 4 | 263 (4.8%) | 197,344 (18.3%) |
|  | 5 (least deprived) | 341 (6.2%) | 193,668 (18.0%) |
|  | Missing | 4,109 (75.1%) | N/A |
|  |  |  |  |
| FSM claimed | Yes (1) | 949 (17.4%) | 196,950 (18.3%) |
|  |  |  |  |
| IDACI/FSM | 1,1 | 74 (1.4%) | 98,115 (9.1%) |
|  | 1,0 | 145 (2.7%) | 158,868 (14.8%) |
|  | 2,1 | 54 (1%) | 50,765 (4.7%) |
|  | 2,0 | 216 (3.9%) | 172,829 (16.1%) |
|  | 3,1 | 30 (0.5%) | 26,684 (2.5%) |
|  | 3,0 | 237 (4.3%) | 178,037 (16.5%) |
|  | 4,1 | * | 14,527 (1.3%) |
|  | 4,0 | * | 182,817 (17.0%) |
|  | 5,1 | * | 6,859 (0.6%) |
|  | 5,0 | * | 186,809 (17.4%) |
|  | Missing | 4,109 (75.1%) | N/A |
|  |  |  |  |
| Region | East Midlands | 335 (6.1%) | 93,762 (8.7%) |
|  | East of England | 702 (12.8%) | 122,824 (11.4%) |
|  | London | 1,032 (18.9%) | 153,684 (14.3%) |
|  | North East | 158 (2.9%) | 52,236 (4.9%) |
|  | North West | 788 (14.4%) | 148,375 (13.8%) |
|  | South East | 894 (16.3%) | 171,479 (15.9%) |
|  | South West | 579 (10.6%) | 103,395 (9.6%) |
|  | West Midlands | 641 (11.7%) | 120,949 (11.2%) |
|  | Yorkshire & The Humber | 340 (6.2%) | 109,606 (10.2%) |
|  |  |  |  |
| Ever SEND (primary school) |  | 2,827 (51.7%) | 390,183 (36.3%) |
|  |  |  |  |
| Highest ever SEND (primary school) | None | 2,642 (48.3%) | 686,127 (63.7%) |
|  | AAPS | 1,275 (23.3%) | 354,684 (33.0%) |
|  | SEHCP | 1,552 (28.4%) | 35,499 (3.3%) |
|  |  |  |  |
| AP/PRU (year 7) |  | 1,765 (32.3%) | 1,618 (0.2%) |
|  |  |  |  |
| Special school (year 7) |  | 129 (2.4%) | 16,529 (1.5%) |
|  |  |  |  |

^a^ Row percentages. All other percentages are column percentages. * Suppressed due to low cell counts. AAPS Action, Action Plus or Support; AP/PRU Alternative provision / Pupil Referral Unit; CiN child in need; CLA child looked after; CPP child protection plan; CSC children’s social care; FSM free school meals; IDACI income domain affecting children index; SEHCP statement or Education, Health & Care Plan; SEND special educational needs and disabilities.
