# Supplementary material for: Evaluation of pushing out of children from all English state schools: Administrative data cohort study of children receiving social care and their peers
Source: Child Abuse Negl. 2022 May;127:105582. doi: 10.1016/j.chiabu.2022.105582 (PMC9077441; doi:10.1016/j.chiabu.2022.105582)
Supplement: Supplementary File 4 — Cohort characteristics. [file mmc4.docx]

## Supplementary File 4: cohort characteristics

Table S4.1. Characteristics of the cohort, disaggregated by inception year

|  |  | Year 7 2011/12 | Year 7 2012/13 | Combined |
| --- | --- | --- | --- | --- |
|  |  | n (%) | n (%) | n (%) |
| n |  | 543,084 | 533,226 | 1,076,310 |
| Exposure (yr 4 to 6) | None | 502,448 (92.5%) | 488,670 (91.6%) | 991,118 (92.1%) |
|  | CiN | 35,525 (6.5%) | 37,845 (7.1%) | 73,370 (6.8%) |
|  | CPP | 2,134 (0.4%) | 3,554 (0.7%) | 5,688 (0.5%) |
|  | CLA | 2,977 (0.5%) | 3,157 (0.6%) | 6,134 (0.6%) |
|  |  |  |  |  |
| Gender | Male | 277,936 (51.2%) | 272,824 (51.2%) | 550,760 (51.2%) |
|  | Female | 265,148 (48.8%) | 260,402 (48.8%) | 525,550 (48.8%) |
|  |  |  |  |  |
| Ethnicity | White | 434,492 (80%) | 421,826 (79.1%) | 856,318 (79.6%) |
|  | Black | 27,384 (5.0%) | 27,759 (5.2%) | 55,143 (5.1%) |
|  | Mixed | 22,232 (4.1%) | 22,837 (4.3%) | 45,069 (4.2%) |
|  | Asian | 51,450 (9.5%) | 53,069 (10.0%) | 10,4519 (9.7%) |
|  | Other | 7,526 (1.4%) | 7,735 (1.5%) | 15,261 (1.4%) |
|  |  |  |  |  |
| Language | English | 460,153 (84.7%) | 446,173 (83.7%) | 906,326 (84.2%) |
|  | Other | 82,931 (15.3%) | 87,053 (16.3%) | 169,984 (15.8%) |
|  |  |  |  |  |
| IDACI fifths | 1 (most deprived) | 128,800 (23.7%) | 128,183 (24.0%) | 256,983 (23.9%) |
|  | 2 | 112,635 (20.7%) | 110,959 (20.8%) | 223,594 (20.8%) |
|  | 3 | 103,671 (19.1%) | 101,050 (19.0%) | 204,721 (19.0%) |
|  | 4 | 99,908 (18.4%) | 97,436 (18.3%) | 197,344 (18.3%) |
|  | 5 (least deprived) | 98,070 (18.1%) | 95,598 (17.9%) | 193,668 (18.0%) |
|  |  |  |  |  |
| FSM claimed | Yes (1) | 99,331 (18.3%) | 97,619 (18.3%) | 196,950 (18.3%) |
|  |  |  |  |  |
| IDACI/FSM | 1,1 | 49,473 (9.1%) | 48,642 (9.1%) | 98,115 (9.1%) |
|  | 1,0 | 79,327 (14.6%) | 79,541 (14.9%) | 158,868 (14.8%) |
|  | 2,1 | 25,481 (4.7%) | 25,284 (4.7%) | 50,765 (4.7%) |
|  | 2,0 | 87,154 (16%) | 85,675 (16.1%) | 172,829 (16.1%) |
|  | 3,1 | 13,484 (2.5%) | 13,200 (2.5%) | 26,684 (2.5%) |
|  | 3,0 | 90,187 (16.6%) | 87,850 (16.5%) | 178,037 (16.5%) |
|  | 4,1 | 7,276 (1.3%) | 7,251 (1.4%) | 14,527 (1.3%) |
|  | 4,0 | 92,632 (17.1%) | 90,185 (16.9%) | 182,817 (17%) |
|  | 5,1 | 3,617 (0.7%) | 3,242 (0.6%) | 6,859 (0.6%) |
|  | 5,0 | 94,453 (17.4%) | 92,356 (17.3%) | 186,809 (17.4%) |
|  |  |  |  |  |
| Region | East Midlands | 47,654 (8.8%) | 46,108 (8.6%) | 93,762 (8.7%) |
|  | East of England | 61,715 (11.4%) | 61,109 (11.5%) | 122,824 (11.4%) |
|  | London | 77,224 (14.2%) | 76,460 (14.3%) | 153,684 (14.3%) |
|  | North East | 26,455 (4.9%) | 25,781 (4.8%) | 52,236 (4.9%) |
|  | North West | 74,791 (13.8%) | 73,584 (13.8%) | 148,375 (13.8%) |
|  | South East | 86,414 (15.9%) | 85,065 (16.0%) | 171,479 (15.9%) |
|  | South West | 52,563 (9.7%) | 50,832 (9.5%) | 103,395 (9.6%) |
|  | West Midlands | 61,082 (11.2%) | 59,867 (11.2%) | 120,949 (11.2%) |
|  | Yorkshire & The Humber | 55,186 (10.2%) | 54,420 (10.2%) | 109,606 (10.2%) |
|  |  |  |  |  |
| Ever SEND | (primary school) | 195,226 (35.9%) | 194,957 (36.6%) | 390,183 (36.3%) |
|  |  |  |  |  |
| Highest ever SEND | None | 347,858 (64.1%) | 338,269 (63.4%) | 686,127 (63.7%) |
| (primary school) | AAPS | 177,231 (32.6%) | 177,453 (33.3%) | 354,684 (33.0%) |
|  | SEHCP | 17,995 (3.3%) | 17,504 (3.3%) | 35,499 (3.3%) |
|  |  |  |  |  |
| AP/PRU (year 7) |  | 812 (0.1%) | 806 (0.2%) | 1,618 (0.2%) |
|  |  |  |  |  |
| Special school (year 7) |  | 8,520 (1.6%) | 8,009 (1.5%) | 16,529 (1.5%) |
|  |  |  |  |  |

AAPS Action, Action Plus or Support; AP/PRU Alternative provision / Pupil Referral Unit; CiN child in need; CLA child looked after; CPP child protection plan; FSM free school meals; IDACI income domain affecting children index; SEHCP statement or Education, Health & Care Plan; SEND special educational needs and disabilities.
