# Supplementary material for: Evaluation of pushing out of children from all English state schools: Administrative data cohort study of children receiving social care and their peers
Source: Child Abuse Negl. 2022 May;127:105582. doi: 10.1016/j.chiabu.2022.105582 (PMC9077441; doi:10.1016/j.chiabu.2022.105582)
Supplement: Supplementary File 5 — Annual and cumulative proportions of non-enrolment by all variables. [file mmc5.docx]

## Supplementary File 5: annual and cumulative proportions of non-enrolment by all variables

Table S5.1. Annual and cumulative proportions of non-enrolment (children in mainstream settings in year 7, n = 1,059,781)

|  |  | Annual | | | |  | Cumulative | | | |
| --- | --- | --- | --- | --- | --- | --- | --- | --- | --- | --- |
|  |  | Yr 8 | Yr 9 | Yr 10 | Yr 11 |  | Yr 8 | Yr 9 | Yr 10 | Yr 11 |
|  |  | n (%) | n (%) | n (%) | n (%) |  | n (%) | n (%) | n (%) | n (%) |
|  |  |  |  |  |  |  |  |  |  |  |
| All children |  | 10,189 (1.0%) | 18,332 (1.7%) | 25,472 (2.4%) | 35,913 (3.4%) |  | 10,189 (1.0%) | 20,593 (1.9%) | 30,849 (2.9%) | 44,049 (4.2%) |
|  |  |  |  |  |  |  |  |  |  |  |
| CSC exposure  (yr 4 to 6) | None | 9,196 (0.9%) | 16,654 (1.7%) | 22,809 (2.3%) | 31,222 (3.2%) |  | 9,196 (0.9%) | 18,472 (1.9%) | 27,158 (2.8%) | 37,678 (3.8%) |
|  | CiN | 824 (1.3%) | 1,418 (2.2%) | 2,241 (3.4%) | 3,941 (6.0%) |  | 824 (1.3%) | 1777 (2.7%) | 3,093 (4.7%) | 5,319 (8.1%) |
|  | CPP | 84 (1.6%) | 128 (2.5%) | 204 (3.9%) | 396 (7.6%) |  | 84 (1.6%) | 169 (3.2%) | 291 (5.6%) | 542 (10.4%) |
|  | CLA | 85 (1.6%) | 132 (2.4%) | 218 (4.0%) | 354 (6.5%) |  | 85 (1.6%) | 175 (3.2%) | 307 (5.6%) | 510 (9.4%) |
|  |  |  |  |  |  |  |  |  |  |  |
| Gender | Male | 5,288 (1.0%) | 9,305 (1.7%) | 12,755 (2.4%) | 18,365 (3.4%) |  | 5,288 (1.0%) | 10,545 (1.7%) | 15,627 (2.6%) | 22,720 (4.2%) |
|  | Female | 4,901 (0.9%) | 9,027 (1.7%) | 12,717 (2.4%) | 17,548 (3.4%) |  | 4,901 (0.9%) | 10,048 (1.9%) | 15,222 (2.9%) | 21,329 (4.1%) |
|  |  |  |  |  |  |  |  |  |  |  |
| Ethnicity | White | 6,634 (0.8%) | 12,623 (1.5%) | 18,542 (2.2%) | 27,491 (3.3%) |  | 6,634 (0.8%) | 14,058 (1.7%) | 22,026 (2.6%) | 32,847 (3.9%) |
|  | Black | 1,022 (1.9%) | 1,631 (3.0%) | 1,961 (3.6%) | 2,472 (4.6%) |  | 1,022 (1.9%) | 1,917 (3.5%) | 2,621 (4.8%) | 3,432 (6.3%) |
|  | Mixed | 555 (1.3%) | 982 (2.2%) | 1,313 (3.0%) | 1,834 (4.1%) |  | 555 (1.3%) | 1,140 (2.6%) | 1,678 (3.8%) | 2,404 (5.4%) |
|  | Asian | 1,438 (1.4%) | 2,270 (2.2%) | 2,650 (2.6%) | 2,959 (2.9%) |  | 1,438 (1.4%) | 2,565 (2.5%) | 3,338 (3.2%) | 3,945 (3.8%) |
|  | Other | 540 (3.6%) | 826 (5.5%) | 1,006 (6.7%) | 1,157 (7.7%) |  | 540 (3.6%) | 913 (6.1%) | 1,186 (7.9%) | 1,421 (9.4%) |
|  |  |  |  |  |  |  |  |  |  |  |
| First language | English | 6,590 (0.7%) | 12,711 (1.4%) | 18,702 (2.1%) | 27,793 (3.1%) |  | 6,590 (0.7%) | 14,248 (1.6%) | 22,513 (2.5%) | 33,707 (3.8%) |
|  | Other | 3,599 (2.1%) | 5,621 (3.3%) | 6,770 (4.0%) | 8,120 (4.8%) |  | 3,599 (2.1%) | 6,345 (3.8%) | 8,336 (5.0%) | 10,342 (6.2%) |
|  |  |  |  |  |  |  |  |  |  |  |
| IDACI fifths  (year 7) | 1 (most deprived) | 2,546 (1.0%) | 4,279 (1.7%) | 6,348 (2.5%) | 10,230 (4.1%) |  | 2,546 (1.0%) | 5,105 (2.0%) | 8,275 (3.3%) | 13,285 (5.3%) |
|  | 2 | 2,160 (1.0%) | 3,717 (1.7%) | 5,147 (2.3%) | 7,686 (3.5%) |  | 2,160 (1.0%) | 4,283 (1.9%) | 6,485 (3.0%) | 9,684 (4.4%) |
|  | 3 | 1,859 (0.9%) | 3,370 (1.7%) | 4,605 (2.3%) | 6,360 (3.2%) |  | 1,859 (0.9%) | 3,745 (1.9%) | 5,514 (2.7%) | 7,720 (3.8%) |
|  | 4 | 1,728 (0.9%) | 3,264 (1.7%) | 4,484 (2.3%) | 5,764 (3.0%) |  | 1,728 (0.9%) | 3,531 (1.8%) | 5,121 (2.6%) | 6,718 (3.4%) |
|  | 5 (least deprived) | 1,896 (1.0%) | 3,702 (1.9%) | 4,888 (2.5%) | 5,873 (3.1%) |  | 1,896 (1.0%) | 3,929 (2.0%) | 5,454 (2.8%) | 6,642 (3.5%) |
|  |  |  |  |  |  |  |  |  |  |  |
| FSM claimed  (year 7) | No (0) | 7,918 (0.9%) | 14,502 (1.7%) | 19,674 (2.3%) | 25,909 (3.0%) |  | 7,918 (0.9%) | 15,892 (1.8%) | 23,035 (2.7%) | 30,944 (3.6%) |
|  | Yes (1) | 2,271 (1.2%) | 3,830 (2.0%) | 5,798 (3.0%) | 10,004 (5.2%) |  | 2,271 (1.2%) | 4,701 (2.5%) | 7,814 (4.1%) | 13,105 (6.9%) |
|  |  |  |  |  |  |  |  |  |  |  |
| IDACI/FSM  (year 7) | 1,1 | 1,063 (1.1%) | 1,786 (1.9%) | 2,842 (3.0%) | 5,062 (5.3%) |  | 1,063 (1.1%) | 2,243 (2.4%) | 3,878 (4.1%) | 6,715 (7.1%) |
|  | 1,0 | 1,483 (0.9%) | 2,493 (1.6%) | 3,506 (2.2%) | 5,168 (3.3%) |  | 1,483 (0.9%) | 2,862 (1.8%) | 4,397 (2.8%) | 6,570 (4.2%) |
|  | 2,1 | 627 (1.3%) | 1,016 (2.1%) | 1,524 (3.1%) | 2,610 (5.3%) |  | 627 (1.3%) | 1,246 (2.5%) | 2,052 (4.2%) | 3,414 (6.9%) |
|  | 2,0 | 1,533 (0.9%) | 2,701 (1.6%) | 3,623 (2.1%) | 5,076 (3.0%) |  | 1,533 (0.9%) | 3,037 (1.8%) | 4,433 (2.6%) | 6,270 (3.7%) |
|  | 3,1 | 317 (1.2%) | 559 (2.2%) | 795 (3.1%) | 1,327 (5.1%) |  | 317 (1.2%) | 673 (2.6%) | 1,061 (4.1%) | 1,704 (6.6%) |
|  | 3,0 | 1,542 (0.9%) | 2,811 (1.6%) | 3,810 (2.2%) | 5,033 (2.9%) |  | 1,542 (0.9%) | 3,072 (1.7%) | 4,453 (2.5%) | 6,016 (3.4%) |
|  | 4,1 | 178 (1.3%) | 313 (2.2%) | 439 (3.1%) | 695 (4.9%) |  | 178 (1.3%) | 364 (2.6%) | 570 (4.1%) | 886 (6.3%) |
|  | 4,0 | 1,550 (0.9%) | 2,951 (1.6%) | 4,045 (2.2%) | 5,069 (2.8%) |  | 1,550 (0.9%) | 3,167 (1.8%) | 4,551 (2.5%) | 5,832 (3.2%) |
|  | 5,1 | 86 (1.3%) | 156 (2.3%) | 198 (3.0%) | 310 (4.7%) |  | 86 (1.3%) | 175 (2.6%) | 253 (3.8%) | 386 (5.8%) |
|  | 5,0 | 1,810 (1.0%) | 3,546 (1.9%) | 4,690 (2.5%) | 5,563 (3.0%) |  | 1,810 (1.0%) | 3,754 (2.0%) | 5,201 (2.8%) | 6,256 (3.4%) |
|  |  |  |  |  |  |  |  |  |  |  |
| Region | East Midlands | 802 (0.9%) | 1,452 (1.6%) | 2,029 (2.2%) | 2,982 (3.2%) |  | 802 (0.9%) | 1,618 (1.7%) | 2,417 (2.6%) | 3,571 (3.9%) |
|  | East of England | 1,104 (0.9%) | 2,102 (1.7%) | 2,824 (2.3%) | 4,121 (3.4%) |  | 1,104 (0.9%) | 2,320 (1.9%) | 3,382 (2.8%) | 4,901 (4.0%) |
|  | London | 2,116 (1.4%) | 3,680 (2.4%) | 4,761 (3.1%) | 6,481 (4.3%) |  | 2,116 (1.4%) | 4,173 (2.8%) | 5,948 (3.9%) | 8,320 (5.5%) |
|  | North East | 355 (0.7%) | 604 (1.2%) | 879 (1.7%) | 1,340 (2.6%) |  | 355 (0.7%) | 688 (1.3%) | 1,070 (2.1%) | 1,616 (3.2%) |
|  | North West | 1,197 (0.8%) | 1,954 (1.3%) | 2,840 (1.9%) | 4,035 (2.8%) |  | 1,197 (0.8%) | 2,273 (1.6%) | 3,556 (2.4%) | 5,193 (3.6%) |
|  | South East | 1,826 (1.1%) | 3,401 (2%) | 4,642 (2.8%) | 6,373 (3.8%) |  | 1,826 (1.1%) | 3,740 (2.2%) | 5,463 (3.2%) | 7,570 (4.5%) |
|  | South West | 925 (0.9%) | 1,800 (1.8%) | 2,564 (2.5%) | 3,547 (3.5%) |  | 925 (0.9%) | 1,986 (1.9%) | 2,983 (2.9%) | 4,177 (4.1%) |
|  | West Midlands | 977 (0.8%) | 1,807 (1.5%) | 2,417 (2.0%) | 3,526 (3.0%) |  | 977 (0.8%) | 2,036 (1.7%) | 3,002 (2.5%) | 4,403 (3.7%) |
|  | Yorkshire & The Humber | 887 (0.8%) | 1,532 (1.4%) | 2,516 (2.3%) | 3,508 (3.2%) |  | 887 (0.8%) | 1,759 (1.6%) | 3,028 (2.8%) | 4,298 (4.0%) |
|  |  |  |  |  |  |  |  |  |  |  |
| Ever SEND  (primary school) | No | 6,431 (0.9%) | 11,779 (1.7%) | 15,798 (2.3%) | 20,090 (2.9%) |  | 6,431 (0.9%) | 12,827 (1.9%) | 18,300 (2.7%) | 23,756 (3.5%) |
|  | Yes | 3758 (1.0%) | 6,553 (1.8%) | 9,674 (2.6%) | 15,823 (4.2%) |  | 3,758 (1.0%) | 7,766 (2.1%) | 12,549 (3.4%) | 20,293 (5.4%) |
|  |  |  |  |  |  |  |  |  |  |  |
| Highest ever SEND  (primary school) | None | 6,431 (0.9%) | 11,779 (1.7%) | 15,798 (2.3%) | 20,090 (2.9%) |  | 6,431 (0.9%) | 12,827 (1.9%) | 18,300 (2.7%) | 23,756 (3.5%) |
|  | AAPS | 3,576 (1.0%) | 6,239 (1.8%) | 9,233 (2.6%) | 15,104 (4.3%) |  | 3,576 (1.0%) | 7,369 (2.1%) | 11,917 (3.4%) | 19,265 (5.4%) |
|  | SEHCP | 182 (0.9%) | 314 (1.6%) | 441 (2.2%) | 719 (3.6%) |  | 182 (0.9%) | 397 (2.0%) | 632 (3.2%) | 1,028 (5.1%) |
|  |  |  |  |  |  |  |  |  |  |  |
| AP/PRU  (primary school to yr 6) | No | 10,151 (1%) | 18,280 (1.7%) | 25,402 (2.4%) | 35,815 (3.4%) |  | 10,151 (1.0%) | 20,521 (1.9%) | 30,740 (2.9%) | 43,896 (4.1%) |
|  | Yes | 38 (4.8%) | 52 (6.5%) | 70 (8.8%) | 98 (12.3%) |  | 38  (4.8%) | 72  (9.0%) | 109  (13.7%) | 153  (19.2%) |
|  |  |  |  |  |  |  |  |  |  |  |

AAPS Action, Action Plus or Support; AP/PRU Alternative provision / Pupil Referral Unit; CiN child in need; CLA child looked after; CPP child protection plan; CSC children’s social care; FSM free school meals; IDACI income deprivation affecting children index; SEHCP statement or Education, Health & Care Plan; SEND special educational needs and disabilities; yr year.
