# Supplementary material for: Evaluation of pushing out of children from all English state schools: Administrative data cohort study of children receiving social care and their peers
Source: Child Abuse Negl. 2022 May;127:105582. doi: 10.1016/j.chiabu.2022.105582 (PMC9077441; doi:10.1016/j.chiabu.2022.105582)
Supplement: Supplementary File 6 — Full modelling results. [file mmc6.docx]

## Supplementary File 6: Full modelling results

Table S6.1. Coefficients and standard errors from hierarchical logistic regression models of non-enrolment in years 10/11 of children in the mainstream cohort

|  |  | Univariable  models | 1 | 2 | 3 | 4 | 5 |
| --- | --- | --- | --- | --- | --- | --- | --- |
|  |  |  | (null) | Coef (SE) | Coef (SE) | Coef (SE) | Coef (SE) |
| Intercept |  |  |  | -3.75 (0.05) | -3.96 (0.05) | -3.95 (0.06) | -3.65 (0.05) |
| *Individual-level variables* | | | | | | | |
| CSC exposure | None | Ref | - | Reference | Reference | Reference | Reference |
| (yr 4 to 9) | CiN | 0.93 (0.01) |  | 0.93 (0.01) | 0.8 (0.02) | 0.74 (0.03) | 0.60 (0.03) |
|  | CPP | 1.28 (0.03) |  | 1.28 (0.03) | 1.12 (0.03) | 1.00 (0.06) | 0.80 (0.06) |
|  | CLA | 1.56 (0.03) |  | 1.56 (0.03) | 1.35 (0.03) | 1.52 (0.06) | 1.34 (0.06) |
| SEND to yr 9 |  | - |  |  | 0.49 (0.01) | 0.48 (0.01) | 0.40 (0.01) |
| Interaction | CiN & SEND | - |  |  |  | 0.08 (0.03) | 0.09 (0.03) |
|  | CPP & SEND | - |  |  |  | 0.13 (0.06) | 0.11 (0.07) |
|  | CLA & SEND | - |  |  |  | -0.20 (0.06) | -0.18 (0.07) |
| Female |  | -0.05 (0.01) |  |  |  |  | 0.02 (0.01) |
| Ethnicity | White | Ref |  |  |  |  | Ref |
|  | Black | 0.21 (0.02) |  |  |  |  | -0.22 (0.03) |
|  | Mixed | 0.20 (0.02) |  |  |  |  | 0.06 (0.03) |
|  | Asian | -0.30 (0.02) |  |  |  |  | -0.88 (0.03) |
|  | Other | 0.82 (0.03) |  |  |  |  | 0.21 (0.04) |
| First language not English |  | 0.49 (0.02) |  |  |  |  | 0.88 (0.02) |
| IDACI/FSM | 1,1 | Ref |  |  |  |  | Ref |
|  | 1,0 | -0.58 (0.02) |  |  |  |  | -0.34 (0.01) |
|  | 2,1 | -0.04 (0.02) |  |  |  |  | 0.01 (0.02) |
|  | 2,0 | -0.78 (0.02) |  |  |  |  | -0.43 (0.02) |
|  | 3,1 | -0.15 (0.03) |  |  |  |  | -0.06 (0.02) |
|  | 3,0 | -0.88 (0.02) |  |  |  |  | -0.46 (0.03) |
|  | 4,1 | -0.19 (0.04) |  |  |  |  | -0.05 (0.02) |
|  | 4,0 | -1.00 (0.02) |  |  |  |  | -0.53 (0.04) |
|  | 5,1 | -0.26 (0.05) |  |  |  |  | -0.08 (0.02) |
|  | 5,0 | -1.00 (0.02) |  |  |  |  | -0.49 (0.06) |
| Ever AP/PRU to yr 9 |  | 1.65 (0.03) |  |  |  |  | 0.96 (0.04) |
|  |  |  |  |  |  |  |  |
| *Variance components* | | | | | | | |
| Level 2 (LA) SD |  | - | 0.24 | 0.22 | 0.22 | 0.22 | 0.22 |
| % explained |  | - | - | 8.3% | 8.3% | 8.3% | 8.3% |
|  |  |  |  |  |  |  |  |
| Level 3 (region) SD |  | - | 0.18 | 0.19 | 0.18 | 0.18 | 0.15 |
| % explained |  | - | - | -5.6% | 0.0% | 0.0% | 16.7% |
|  |  |  |  |  |  |  |  |
| *Model summaries* | | | | | | | |
| AIC |  | - | 280310 | 274787 | 273104 | 273097 | 268991 |
| LRT p value* |  | - | - | <0.001 | <0.001 | 0.005 | <0.001 |
|  |  |  |  |  |  |  |  |
| *All models* |  |  |  |  |  |  |  |
| n children = 1,059,780; n LAs = 151; n regions = 9 | | | | | | | |
|  |  |  |  |  |  |  |  |

AIC Akaike Information Criterion; AP/PRU alternative provision / Pupil Referral Unit; CiN child in need; CLA child looked after; Coef coefficient; CPP child protection plan; CSC children’s social care; FSM free school meals; IDACI income domain affecting children index; LRT likelihood ratio test; SD standard deviation; SE standard error; SEND special educational needs and disability. * LRTs were conducted against the previous model.
