# Supplementary material for: Evaluation of pushing out of children from all English state schools: Administrative data cohort study of children receiving social care and their peers
Source: Child Abuse Negl. 2022 May;127:105582. doi: 10.1016/j.chiabu.2022.105582 (PMC9077441; doi:10.1016/j.chiabu.2022.105582)
Supplement: Supplementary File 7 — Children in special schools. [file mmc7.docx]

## Supplementary File 7: children in special schools

There were 16,529 children enrolled in special schools in year 7 in 2011/12 or 2012/13 (or aged 11 at the start of those academic years if not following the national curriculum). This Supplementary File contains a parallel analysis of non-enrolment among these children. It is divided into the following sections:

- Cohort characteristics
- Aim one: annual and cumulative incidence of non-enrolment across years 8 to 11
- Aim two: regression modelling of non-enrolment in years 10 and 11

## Cohort characteristics

Table S7.1 shows this cohort’s characteristics.

Table S7.1. Characteristics of the cohort of children in special schools in year 7

|  |  | n (%) |
| --- | --- | --- |
|  |  |  |
| n |  | 16,529 |
|  |  |  |
| Exposure (yr 4 to 6) | None | 7,854 (47.5%) |
|  | CiN | 7,490 (45.3%) |
|  | CPP | 486 (2.9%) |
|  | CLA | 699 (4.2%) |
|  |  |  |
| Exposure (yr 4 to 9) | None | 6,221 (37.6%) |
|  | CiN | 8,258 (50.0%) |
|  | CPP | 722 (4.4%) |
|  | CLA | 1,328 (8.0%) |
|  |  |  |
| Female | Male | 4,605 (27.9%) |
|  |  |  |
| Ethnicity | White | 13,313 (80.5%) |
|  | Black | 948 (5.7%) |
|  | Mixed | 723 (4.4%) |
|  | Asian | 1,333 (8.1%) |
|  | Other | 212 (1.3%) |
|  |  |  |
| First language not English | English | 2,063 (12.5%) |
|  |  |  |
| IDACI fifths | 1 (most deprived) | 5,493 (33.2%) |
|  | 2 | 3,823 (23.1%) |
|  | 3 | 2,950 (17.8%) |
|  | 4 | 2,347 (14.2%) |
|  | 5 (least deprived) | 1,916 (11.6%) |
|  |  |  |
| FSM claimed | Yes (1) | 63,82 (38.6%) |
|  |  |  |
| IDACI/FSM | 1,1 | 3,202 (19.4%) |
|  | 1,0 | 2,291 (13.9%) |
|  | 2,1 | 1621 (9.8%) |
|  | 2,0 | 2,202 (13.3%) |
|  | 3,1 | 862 (5.2%) |
|  | 3,0 | 2,088 (12.6%) |
|  | 4,1 | 481 (2.9%) |
|  | 4,0 | 1,866 (11.3%) |
|  | 5,1 | 216 (1.3%) |
|  | 5,0 | 1,700 (10.3%) |
|  |  |  |
| Region | East Midlands | 1,225 (7.4%) |
|  | East of England | 1,616 (9.8%) |
|  | London | 2,170 (13.1%) |
|  | North East | 969 (5.9%) |
|  | North West | 2,380 (14.4%) |
|  | South East | 3,247 (19.6%) |
|  | South West | 1,320 (8%) |
|  | West Midlands | 2,262 (13.7%) |
|  | Yorkshire & The Humber | 1,340 (8.1%) |
|  |  |  |
| In AP/PRU (primary school to yr 6) |  | 740 (4.5%) |
|  |  |  |
| In AP/PRU (primary school to yr 9) |  | 1,365 (8.3%) |
|  |  |  |

AP/PRU Alternative provision / Pupil Referral Unit; CiN child in need; CLA child looked after; CPP child protection plan; CSC children’s social care; FSM free school meals; IDACI income deprivation affecting children index; yr year

## Aim one: annual and cumulative proportions of non-enrolment across years 8 to 11

Table S7.2 presents the annual and cumulative proportions of non-enrolment across years 8 to 11. As with children in mainstream settings in year 7, children’s social care (CSC)-exposed children had a higher risk of non-enrolment, annually and cumulatively compared to non-exposed children.

Table S7.2. Annual and cumulative proportion of non-enrolment among children in special schools in year 7 (n =16,529)

|  |  | Annual | | | |  | Cumulative | | | |
| --- | --- | --- | --- | --- | --- | --- | --- | --- | --- | --- |
|  |  | Yr 8 | Yr 9 | Yr 10 | Yr 11 |  | Yr 8 | Yr 9 | Yr 10 | Yr 11 |
|  |  | n (%) | n (%) | n (%) | n (%) |  | n (%) | n (%) | n (%) | n (%) |
|  |  |  |  |  |  |  |  |  |  |  |
| All children |  | 154 (0.9%) | 240 (1.5%) | 376 (2.3%) | 554 (3.3%) |  | 154 (0.9%) | 301 (1.8%) | 500 (3.0%) | 763 (4.6%) |
|  |  |  |  |  |  |  |  |  |  |  |
| CSC exposure  (yr 4 to 6) | None | 56 (0.7%) | 78 (1.0%) | 119 (1.5%) | 189 (2.4%) |  | 56 (0.7%) | 100 (1.3%) | 163 (2.1%) | 258 (3.3%) |
|  | CiN | * | * | 206 (2.8%) | 287 (3.8%) |  | 79 (1.1%) | 160 (2.1%) | 270 (3.6%) | 395 (5.3%) |
|  | CPP | * | * | 15 (3.1%) | 32 (6.6%) |  | * | * | 19 (3.9%) | 40 (8.2%) |
|  | CLA | 13 (1.9%) | 27 (3.9%) | 36 (5.2%) | 46 (6.6%) |  | * | * | 48 (6.9%) | 70 (10.0%) |
|  |  |  |  |  |  |  |  |  |  |  |
| Gender | Male | 114 (1.0%) | 176 (1.5%) | 275 (2.3%) | 408 (3.4%) |  | 114 (1.0%) | 222 (1.9%) | 371 (3.1%) | 576 (4.8%) |
|  | Female | 40 (0.9%) | 64 (1.4%) | 101 (2.2%) | 146 (3.2%) |  | 40 (0.9%) | 79 (1.7%) | 129 (2.8%) | 187 (4.1%) |
|  |  |  |  |  |  |  |  |  |  |  |
| Ethnicity | White | 120 (0.9%) | 167 (1.3%) | 279 (2.1%) | 432 (3.2%) |  | 120 (0.9%) | 218 (1.6%) | 374 (2.8%) | 591 (4.4%) |
|  | Black | 11 (1.2%) | 20 (2.1%) | 28 (3.0%) | 37 (3.9%) |  | 11 (1.2%) | 24 (2.5%) | 39 (4.1%) | 54 (5.7%) |
|  | Mixed | * | * | 21 (2.9%) | 30 (4.1%) |  | * | 24 (3.3%) | 34 (4.7%) | 48 (6.6%) |
|  | Asian | 11 (0.8%) | 23 (1.7%) | 34 (2.6%) | 40 (3.0%) |  | 11 (0.8%) | 23 (1.7%) | 37 (2.8%) | 51 (3.8%) |
|  | Other | * | * | 14 (6.6%) | 15 (7.1%) |  | * | 12 (5.7%) | 16 (7.5%) | 19 (9.0%) |
|  |  |  |  |  |  |  |  |  |  |  |
| First language | English | 132 (0.9%) | 194 (1.3%) | 315 (2.2%) | 470 (3.2%) |  | 132 (0.9%) | 251 (1.7%) | 430 (3.0%) | 662 (4.6%) |
|  | Other | 22 (1.1%) | 46 (2.2%) | 61 (3.0%) | 84 (4.1%) |  | 22 (1.1%) | 50 (2.4%0 | 70 (3.4%) | 101 (4.9%) |
|  |  |  |  |  |  |  |  |  |  |  |
| IDACI fifths  (year 7) | 1 (most deprived) | 51 (0.9%) | 81 (1.5%) | 123 (2.2%) | 212 (3.9%) |  | 51 (0.9%) | 106 (1.9%) | 175 (3.2%) | 288 (5.2%) |
|  | 2 | 30 (0.8%) | 47 (1.2%) | 77 (2.0%) | 124 (3.2%) |  | 30 (0.8%) | 58 (1.5%) | 104 (2.7%) | 172 (4.5%) |
|  | 3 | 30 (1.0%) | 43 (1.5%) | 66 (2.2%) | 82 (2.8%) |  | 30 (1.0%) | 54 (1.8%) | 85 (2.9%) | 121 (4.1%) |
|  | 4 | 20 (0.9%) | 33 (1.4%) | 56 (2.4%) | 67 (2.9%) |  | 20 (0.9%) | 40 (1.7%) | 72 (3.1%) | 95 (4.0%) |
|  | 5 (least deprived) | 23 (1.2%) | 36 (1.9%) | 54 (2.8%) | 69 (3.6%) |  | 23 (1.2%) | 43 (2.2%) | 64 (3.3%) | 87 (4.5%) |
|  |  |  |  |  |  |  |  |  |  |  |
| FSM claimed  (year 7) | No (0) | 97 (1.0%) | 157 (1.5%) | 247 (2.4%) | 348 (3.4%) |  | 97 (1.0%) | 189 (1.9%) | 311 (3.1%) | 464 (4.6%) |
|  | Yes (1) | 57 (0.9%) | 83 (1.3%) | 129 (2.0%) | 206 (3.2%) |  | 57 (0.9%) | 112 (1.8%) | 189 (3.0%) | 299 (4.7%) |
|  |  |  |  |  |  |  |  |  |  |  |
| IDACI/FSM  (year 7) | 1,1 | 28 (0.9%) | 42 (1.3%) | 63 (2.0%) | 108 (3.4%) |  | 28 (0.9%) | 56 (1.7%) | 94 (2.9%) | 152 (4.7%) |
|  | 1,0 | 23 (1.0%) | 39 (1.7%) | 60 (2.6%) | 104 (4.5%) |  | 23 (1.0%) | 50 (2.2%) | 81 (3.5%) | 136 (5.9%) |
|  | 2,1 | 10 (0.6%) | 18 (1.1%) | 29 (1.8%) | 53 (3.3%) |  | 10 (0.6%) | 23 (1.4%) | 42 (2.6%) | 73 (4.5%) |
|  | 2,0 | 20 (0.9%) | 29 (1.3%) | 48 (2.2%) | 71 (3.2%) |  | 20 (0.9%) | 35 (1.6%) | 62 (2.8%) | 99 (4.5%) |
|  | 3,1 | 11 (1.3%) | 10 (1.2%) | 16 (1.9%) | 23 (2.7%) |  | 11 (1.3%) | 16 (1.9%) | 25 (2.9%) | 37 (4.3%) |
|  | 3,0 | 19 (0.9%) | 33 (1.6%) | 50 (2.4%) | 59 (2.8%) |  | 19 (0.9%) | 38 (1.8%) | 60 (2.9%) | 84 (4.0%) |
|  | 4,1 | * | * | * | 12 (2.5%) |  | * | 10 (2.1%) | 18 (3.7%) | 23 (4.8%) |
|  | 4,0 | * | * | * | 55 (2.9%) |  | * | 30 (1.6%) | 54 (2.8%) | 72 (3.9%) |
|  | 5,1 | * | * | * | 10 (4.6%) |  | * | * | 10 (4.6%) | 14 (6.5%) |
|  | 5,0 | * | * | * | 59 (3.5%) |  | * | * | 54 (3.2%) | 73 (4.3%) |
|  |  |  |  |  |  |  |  |  |  |  |
| Region | East Midlands | 10 (0.8%) | 13 (1.1%) | 22 (1.8%) | 29 (2.4%) |  | 10 (0.8%) | 17 (1.4%) | 29 (2.4%) | 43 (3.5%) |
|  | East of England | 14 (0.9%) | 21 (1.3%) | 31 (1.9%) | 56 (3.5%) |  | 14 (0.9%) | 26 (1.6%) | 40 (2.5%) | 72 (4.5%) |
|  | London | 22 (1.0%) | 40 (1.8%) | 51 (2.4%) | 71 (3.3%) |  | 22 (1.0%) | 50 (2.3%) | 73 (3.4%) | 105 (4.8%) |
|  | North East | * | 13 (1.3%) | 24 (2.5%) | 37 (3.8%) |  | * | 15 (1.5%) | 29 (3.0%) | 46 (4.7%) |
|  | North West | 24 (1.0%) | 25 (1.1%) | 56 (2.4%) | 84 (3.5%) |  | 24 (1.0%) | 38 (1.6%) | 74 (3.1%) | 113 (4.7%) |
|  | South East | 35 (1.1%) | 67 (2.1%) | 86 (2.6%) | 112 (3.4%) |  | 35 (1.1%) | 80 (2.5%) | 115 (3.5%) | 161 (5.0%) |
|  | South West | 11 (0.8%) | 17 (1.3%) | 30 (2.3%) | 42 (3.2%) |  | 11 (0.8%) | 20 (1.5%) | 39 (3.0%) | 55 (4.2%) |
|  | West Midlands | 24 (1.1%) | 30 (1.3%) | 53 (2.3%) | 68 (3.0%) |  | 24 (1.1%) | 40 (1.8%) | 72 (3.2%) | 102 (4.5%) |
|  | Yorkshire & The Humber | * | 14 (1.0%) | 23 (1.7%) | 55 (4.1%) |  | * | 15 (1.1%) | 29 (2.2%) | 66 (4.9%) |
|  |  |  |  |  |  |  |  |  |  |  |
| AP/PRU  (primary school to yr 6) | No | 137 (0.9%) | 212 (1.3%) | 336 (2.1%) | 500 (3.2%) |  | 137 (0.9%) | 262 (1.7%) | 442 (2.8%) | 676 (4.3%) |
|  | Yes | 17 (2.3%) | 28 (3.8%) | 40 (5.4%) | 54 (7.3%) |  | 17 (2.3%) | 39 (5.3%) | 58  (7.8%) | 87  (11.8%) |
|  |  |  |  |  |  |  |  |  |  |  |

* Suppressed due to low cell counts. AP/PRU Alternative provision / Pupil Referral Unit; CiN child in need; CLA child looked after; CPP child protection plan; CSC children’s social care; FSM free school meals; IDACI income domain affecting children index; yr year.

## Aim two: regression modelling of risk factors for non-enrolment in years 10/11

Table S7.3 shows the number and proportions of children in the special schools cohort who: (1) were not enrolled in years 10 or 11; (2) sat <5 GCSE or equivalent exams; and (3) were not enrolled in years 10 or 11 and sat <5 GCSE or equivalent exams. As expected, the vast majority of children in the special schools cohort sat <5 GCSEs. We therefore used the first column as the outcome variable (not enrolled in year 10 or 11), highlighted in bold in Table S7.3, in these analyses. Again, the incidence of non-enrolment was higher among the CSC-exposed children.

Table S7.3. Incidence of off-rolling in years 10 and 11 among the special schools cohort (n = 16,529)

|  |  | Not enrolled yr 8/9 | **Not enrolled yr 10/11** | Sat <5 GCSEs | Not enrolled yr 10/11 & <5 GCSEs or equivalents |
| --- | --- | --- | --- | --- | --- |
|  |  |  | **n (%)** | n (%) | n (%) |
|  |  |  |  |  |  |
| CSC exposure | None | 63 (1.0%) | **154 (2.5%)** | 5,562 (89.4%) | 148 (2.4%) |
| (yr 4 to 9) | CiN | 161 (1.9%) | **311 (3.8%)** | 7,719 (93.5%) | 304 (3.7%) |
|  | CPP | 15 (2.1%) | **54 (7.5%)** | 661 (91.6%) | 54 (7.5%) |
|  | CLA | 62 (4.7%) | **126 (9.5%)** | 1,226 (92.3%) | 125 (9.4%) |
|  |  |  |  |  |  |
| Gender | Male | 222 (1.9%) | **484 (4.1%)** | 10,785 (90.4%) | 473 (4.0%) |
|  | Female | 79 (1.7%) | **161 (3.5%)** | 4,383 (95.2%) | 158 (3.4%) |
|  |  |  |  |  |  |
| Ethnicity | White | 218 (1.6%) | **501 (3.8%)** | 12,123 (91.1%) | 489 (3.7%) |
|  | Black | 24 (2.5%) | **43 (4.5%)** | 902 (95.1%) | 42 (4.4%) |
|  | Mixed | 24 (3.3%) | **36 (5.0%)** | 645 (89.2%) | 35 (4.8%) |
|  | Asian | 23 (1.7%) | **48 (3.6%)** | 1,294 (97.1%) | 48 (3.6%) |
|  | Other | 12 (5.7%) | **17 (8.0%)** | 204 (96.2%) | 17 (8.0%) |
|  |  |  |  |  |  |
| Language | English | 251 (1.7%) | **552 (3.8%)** | 13,186 (91.2%) | 538 (3.7%) |
|  | Other | 50 (2.4%) | **93 (4.5%)** | 1,982 (96.1%) | 93 (4.5%) |
|  |  |  |  |  |  |
| IDACI fifths | 1 (most deprived) | 106 (1.9%) | **238 (4.3%)** | 5,040 (91.8%) | 233 (4.2%) |
|  | 2 | 58 (1.5%) | **146 (3.8%)** | 3,526 (92.2%) | 144 (3.8%) |
|  | 3 | 54 (1.8%) | **102 (3.5%)** | 2,704 (91.7%) | 101 (3.4%) |
|  | 4 | 40 (1.7%) | **81 (3.5%)** | 2,155 (91.8%) | 78 (3.3%) |
|  | 5 (least deprived) | 43 (2.2%) | **78 (4.1%)** | 1,743 (91.0%) | 75 (3.9%) |
|  |  |  |  |  |  |
| FSM | No (0) | 189 (1.9%) | **404 (4.0%)** | 9,292 (91.6%) | 393 (3.9%) |
| claimed | Yes (1) | 112 (1.8%) | **241 (3.8%)** | 5,876 (92.1%) | 238 (3.7%) |
|  |  |  |  |  |  |
| IDACI/FSM | 1,1 (most deprived & claimed FSM) | 56 (1.7%) | **122 (3.8%)** | 2,953 (92.2%) | 119 (3.7%) |
|  | 1,0 | 50 (2.2%) | **116 (5.1%)** | 2,087 (91.1%) | 114 (5.0%) |
|  | 2,1 | 23 (1.4%) | **61 (3.8%)** | 1,503 (92.7%) | 61 (3.8%) |
|  | 2,0 | 35 (1.6%) | **85 (3.9%)** | 2,023 (91.9%) | 83 (3.8%) |
|  | 3,1 | 16 (1.9%) | **28 (3.2%)** | 789 (91.5%) | 28 (3.2%) |
|  | 3,0 | 38 (1.8%) | **74 (3.5%)** | 1,915 (91.7%) | 73 (3.5%) |
|  | 4,1 | * | **18 (3.7%)** | 441 (91.7%) | 18 (3.7%) |
|  | 4,0 | 30 (1.6%) | **63 (3.4%)** | 1,714 (91.9%) | 60 (3.2%) |
|  | 5,1 | * | **12 (5.6%)** | 190 (88.0%) | 12 (5.6%) |
|  | 5,0 (least deprived & no FSM) | 36 (2.1%) | **66 (3.9%)** | 1,553 (91.4%) | 63 (3.7%) |
|  |  |  |  |  |  |
| Region | East Midlands | 17 (1.4%) | **37 (3.0%)** | 1,145 (93.5%) | 35 (2.9%) |
|  | East of England | 26 (1.6%) | **63 (3.9%)** | 1,505 (93.1%) | 63 (3.9%) |
|  | London | 50 (2.3%) | **84 (3.9%)** | 2,067 (95.3%) | 82 (3.8%) |
|  | North East | 15 (1.5%) | **41 (4.2%)** | 868 (89.6%) | 40 (4.1%) |
|  | North West | 38 (1.6%) | **97 (4.1%)** | 2,178 (91.5%) | 97 (4.1%) |
|  | South East | 80 (2.5%) | **133 (4.1%)** | 2,810 (86.5%) | 129 (4.0%) |
|  | South West | 20 (1.5%) | **47 (3.6%)** | 1,257 (95.2%) | 45 (3.4%) |
|  | West Midlands | 40 (1.8%) | **83 (3.7%)** | 2,040 (90.2%) | 81 (3.6%) |
|  | Yorkshire & The Humber | 15 (1.1%) | **60 (4.5%)** | 1,298 (96.9%) | 59 (4.4%) |
|  |  |  |  |  |  |
| AP/PRU | No | 229 (1.5%) | **517 (3.4%)** | 14,020 (92.5%) | 507 (3.3%) |
| (to yr 9) | Yes | 72 (5.3%) | **128 (9.4%)** | 1,148 (84.1%) | 124 (9.1%) |
|  |  |  |  |  |  |

The second column in bold represents the variable used as the outcome in the regression modelling. * suppressed due to small cell counts. AP/PRU Alternative provision / Pupil Referral Unit; CiN child in need; CLA child looked after; CPP child protection plan; CSC children’s social care; FSM free school meals; GCSE General Certificate of Secondary Education; IDACI income deprivation affecting children index; SEHCP statement or Education, Health & Care Plan; SEND special educational needs and disabilities; yr year.

The modelling strategy for children in the special schools cohort was similar to that for those in mainstream settings. The differences were the outcome variable, detailed above, and the inclusion of free school meals (FSM) claimed rather than the combined income deprivation affecting children index (IDACI)/FSM variable due to small numbers. We also did not enter special educational needs and disability (SEND) as all children in special schools have SEND by definition. Finally, attempting to model a three-level model with pupils in LAs in regions resulted in a singular fit, with the region-level variance estimated as zero. We therefore only estimated two level models where pupils were nested in LAs and where the intercept was allowed to vary by LA.

Table S7.4 shows the principal results as odds ratios with 95% confidence intervals. Table S7.5 shows full model results as coefficients with standard errors. CSC exposure was strongly associated with not being enrolled in year 10/11. After adjustments (model 3), the odds of non-enrolment among the CLA group were 3.49 (95% CI 2.71, 4.50) times higher than the non-exposed group; among the CPP group, 3.25 (95% CI 2.33, 4.54) times higher; and among the CiN group, 1.51 (95% CI 1.24, 1.83) times higher.

Table S7.4. Selected odds ratios (OR) and 95% confidence intervals (CI) from hierarchical logistic regression models of non-enrolment in years 10/11 of children in the special schools cohort

|  |  |  | Model | | |
| --- | --- | --- | --- | --- | --- |
|  |  |  | 1 (null) | 2 | 3* |
|  |  |  |  |  |  |
| CSC exposure | None | OR (95% CI) | - | Reference | Reference |
| (yr 4 to 9) | CiN | OR (95% CI) |  | 1.52 (1.25, 1.85) | 1.51 (1.24, 1.83) |
|  | CPP | OR (95% CI) |  | 3.16 (2.31, 4.32) | 3.25 (2.33, 4.54) |
|  | CLA | OR (95% CI) |  | 4.10 (3.24, 5.18) | 3.49 (2.71, 4.50) |
|  |  |  |  |  |  |
| *Variance component* |  |  |  |  |  |
| Level 2 (LA) SD |  |  | 0.31 | 0.28 | 0.27 |
| % explained |  |  | - | 2.7% | 12.9% |
|  |  |  |  |  |  |
| *Model summaries* |  |  |  |  |  |
| AIC |  |  | - | 5324 | 5255 |
| LRT p value† |  |  | - | <0.001 | <0.001 |
|  |  |  |  |  |  |

In all models there were 16,529 children in 148 LAs. * Adjusted for gender, ethnicity, first language, FSM, attendance at AP/PRU to year 9. † LRTs were conducted against the previous model. AIC Akaike Information Criterion; CI confidence interval; CiN child in need; CLA child looked after; CPP child protection plan; CSC children’s social care; LA local authority; LRT likelihood ratio test; OR odds ratio; SD standard deviation.

Table S7.5. Full coefficients and standard errors from hierarchical logistic regression models of non-enrolment in years 10/11 of children in the special schools cohort

|  |  | Model | | | |
| --- | --- | --- | --- | --- | --- |
|  |  | Univariable  models | 1 (null) | 2 | 3* |
| Intercept |  |  |  | -3.69 (0.09) | -3.75 (0.10) |
| *Individual-level variables* | |  |  |  |  |
| CSC exposure | None | Ref | - | Ref | Ref |
| (yr 4 to 9) | CiN | 0.42 (0.10) |  | 0.42 (0.10) | 0.41 (0.10) |
|  | CPP | 1.15 (0.16) |  | 1.15 (0.16) | 1.18 (0.17) |
|  | CLA | 1.41 (0.12) |  | 1.41 (0.12) | 1.25 (0.13) |
| Female |  | -0.15 (0.90) |  |  | -0.12 (0.09) |
| Ethnicity | White | Ref |  |  | Ref |
|  | Black | 0.20 (0.17) |  |  | 0.12 (0.18) |
|  | Mixed | 0.29 (0.17) |  |  | 0.19 (0.18) |
|  | Asian | -0.05 (0.16) |  |  | -0.14 (0.21) |
|  | Other | 0.81 (0.26) |  |  | 0.72 (0.30) |
| First language not English |  | 0.18 (0.12) |  |  | 0.31 (0.17) |
| FSM claimed |  | -0.06 (0.08) |  |  | -0.14 (0.09) |
| Ever AP/PRU to yr 9 |  | 1.09 (0.10) |  |  | 0.86 (0.11) |
|  |  |  |  |  |  |
|  |  |  |  |  |  |
| *Variance components* |  |  |  |  |  |
| Level 2 (LA) SD |  | - | 0.31 | 0.28 | 0.27 |
| % explained |  | - | - | 9.7% | 12.9% |
|  |  |  |  |  |  |
| *Model summaries* |  |  |  |  |  |
| AIC |  | - | 5444 | 5324 | 5255 |
| LRT p value* |  | - | - | <0.001 | <0.001 |
|  |  |  |  |  |  |

In all models there were 16,529 children in 148 LAs. * LRTs were conducted against the previous model. AIC Akaike Information Criterion; CiN child in need; CLA child looked after; Coef coefficient; CPP child protection plan; CSC children’s social care; LA local authority; LRT likelihood ratio test; SD standard deviation; SE standard error; yr year.
